# An Asia-specific variant of human IgG1 represses colorectal tumorigenesis by shaping the tumor microenvironment

**DOI:** 10.1172/JCI153454

**Published:** 2022-03-15

**Authors:** Bing Yang, Zhen Zhang, Xiangjun Chen, Xu-Yan Wang, Shishang Qin, Liaoqi Du, Changjiang Yang, Liyu Zhu, Wenbo Sun, Yongjie Zhu, Qinwen Zheng, Shidong Zhao, Quan Wang, Long Zhao, Yilin Lin, Jinghe Huang, Fan Wu, Lu Lu, Fei Wang, Wenjie Zheng, Xiao-Hua Zhou, Xiaozhen Zhao, Ziye Wang, Sun Xiao-Lin, Yingjiang Ye, Shan Wang, Zhanguo Li, Hai Qi, Zemin Zhang, Dong-Ming Kuang, Lei Zhang, Zhanlong Shen, Wanli Liu

**Affiliations:** 1 School of Life Sciences, Institute for Immunology, MOE Key Laboratory of Protein Sciences, Beijing Advanced Innovation Center for Structural Biology, Collaborative Innovation Center for Diagnosis and Treatment of Infectious Diseases, Beijing Key Lab for Immunological Research on Chronic Diseases, Tsinghua University, Beijing, China.; 2 Department of Gastroenterological Surgery, Laboratory of Surgical Oncology, Beijing Key Laboratory of Colorectal Cancer Diagnosis and Treatment Research, Peking University People’s Hospital, Beijing, China.; 3 MOE Key Laboratory of Gene Function and Regulation, School of Life Sciences, Sun Yat-sen University, Guangzhou, China.; 4 Beijing Advanced Innovation Center for Genomics, Peking-Tsinghua Center for Life Sciences, BIOPIC and School of Life Sciences, Peking University, Beijing, China.; 5 Shanghai Public Health Clinical Center, Key Laboratory of Medical Molecular Virology, School of Basic Medical Sciences and; 6 Shanghai Public Health Clinical Center, Fudan University, Shanghai, China.; 7 Center for Natural Products Research, Chengdu Institute of Biology, Chinese Academy of Sciences, Chengdu, China.; 8 Department of Rheumatology and Clinical Immunology, Peking Union Medical College Hospital, Chinese Academy of Medical Sciences, Peking Union Medical College, The Ministry of Education Key Laboratory, National Clinical Research Center for Dermatologic and Immunologic Diseases, Beijing, China.; 9 School of Public Health, Peking University, Beijing, China.; 10 Department of Rheumatology and Immunology, Beijing Key Laboratory for Rheumatism and Immune Diagnosis (BZ0135), PKU People’s Hospital, Beijing, China.; 11 Department of Basic Medical Sciences, School of Medicine, Institute for Immunology, Tsinghua University, Beijing, China.; 12 Tsinghua-Peking Center for Life Sciences, Beijing, China.

**Keywords:** Immunology, Oncology, Adaptive immunity, Colorectal cancer

## Abstract

Emerging studies have focused on ways to treat cancers by modulating T cell activation. However, whether B cell receptor signaling in the tumor microenvironment (TME) can be harnessed for immunotherapy is unclear. Here, we report that an Asia-specific variant of human IgG1 containing a Gly396 to Arg396 substitution (hIgG1-G396R) conferred improved survival of patients with colorectal cancer (CRC). Mice with knockin of the murine functional homolog mIgG2c-G400R recapitulated the alleviated tumorigenesis and progression in murine colon carcinoma models. Immune profiling of the TME revealed broad mobilizations of IgG1^+^ plasma cells, CD8^+^ T cells, CD103^+^ DCs, and active tertiary lymphoid structure formation, suggesting an effective antitumor microenvironment in hIgG1-G396R CRC patients. Mechanistically, this variant potentiated tumor-associated antigen–specific (TAA-specific) plasma cell differentiation and thus antibody production. These elevated TAA-specific IgG2c antibodies in turn efficiently boosted the antibody-dependent tumor cell phagocytosis and TAA presentation to effector CD8^+^ T cells. Notably, adoptive transfer of TAA-specific class-switched memory B cells harboring this variant exhibited therapeutic efficacy in murine tumor models, indicating their clinical potential. All these results prompted a prospective investigation of hIgG1-G396R in patients with CRC as a biomarker for clinical prognosis and demonstrated that manipulating the functionality of IgG1^+^ memory B cells in tumors could improve immunotherapy outcomes.

## Graphical abstract

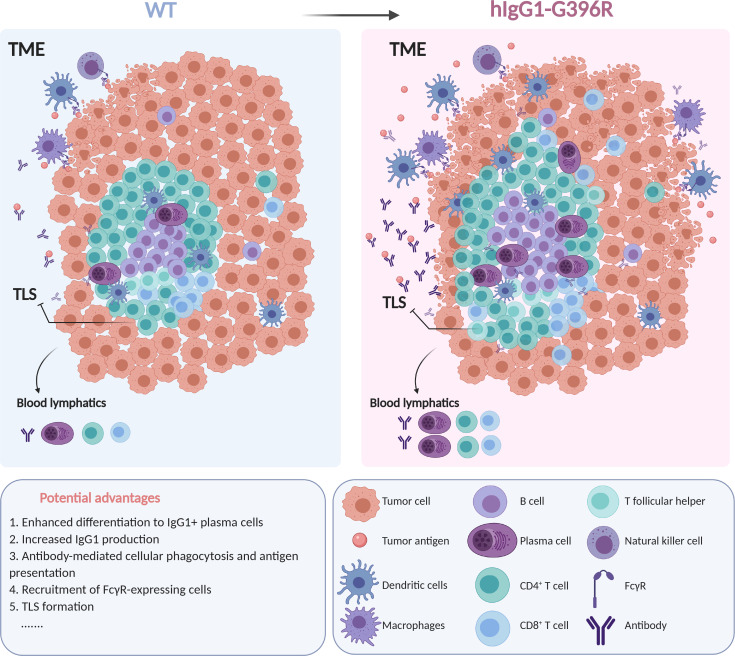

## Introduction

Effective immune responses are indispensable for optimal cancer treatment; however, tumor cells often evade immune clearance by inhibiting neoantigen presentation and inducing immune silencing in the tumor microenvironment (TME) (1). Emerging studies have focused on novel ways to reinvigorate immunosurveillance by modulating T cell activation through T cell receptor engineering or immune checkpoint blockade (2). However, the response rate of immunotherapy still needs to be improved based on the current paradigms (3, 4). Thus, investigations on other immune cells in the TME are required to replenish the immunotherapy toolbox for improved outcomes. Tumor-infiltrating B lymphocytes have been indicated as a predictor of increased patient survival with documented negative regulation of tumor progression in breast cancer, melanoma, colorectal cancer, et al. (5–8). Recent investigations of immune profiles in the TME have identified tertiary lymphoid structures (TLSs) as favorable prognostic markers in several cancer types, and B cells have been shown to be one of the major initiators of TLS formation (9–12). These recent studies indicate the potential of manipulating B cell antigen receptor (BCR) signaling in immunosurveillance against tumors.

Within the TME, tumor-associated antigens (TAAs), including neoantigens, can drive tumor-specific B cells to undergo immune activation, class switching to IgG1^+^ memory B cells, and differentiation to IgG1 antibody–secreting plasma cells (13). TAA-specific IgG1 antibodies can rapidly target tumor cells for attack by innate immune cells through the binding of IgG fragment crystallizable (Fc) with activating Fcγ receptor (FcγR) (14), including antibody-dependent cytotoxicity (ADCC) and phagocytosis (ADCP) (15). These steps can also enhance tumor antigen presentation by dendritic cells (DCs) for broad immune mobilization (16). Thus, class-switched IgG1^+^ memory B cell activation, proliferation, and subsequent differentiation to plasma cells should be considered as cores to harness B cells in antitumor responses. As the initiation of IgG1/BCR signaling is critical for IgG memory antibody response, a leading opinion is that the intracellular tail of membrane-bound IgG (denoted mIgG-tail) is the core molecular component of immunological memory. We recently reported that a single-nucleotide polymorphism (SNP), rs117518546 (C>T, glycine>arginine; hIgG1-G396R), located in the mIgG-tail–coding region of the human immunoglobulin heavy constant γ 1 gene (*IGHG1*), enhances IgG1/BCR signaling and positively correlates with systemic lupus erythematosus (17).

In this report, we investigated whether hIgG1-G396R could modulate cancer progression and survival by focusing on a potential role in colorectal cancer (CRC), which is one of the most common causes of cancer-related deaths and a complex disease with great heterogeneity caused by both genetic and environmental risk factors (18). We identified a protective role of the hIgG1-G396R variant for tumor progression and survival in CRC patients, which was readily recapitulated in multiple murine tumor models. As the underlying mechanism, we found enhanced TAA-specific plasma cell differentiation and antibody production in both variant-harboring CRC patients and genetic knockin mice. The potentiated TAA-specific antibody in turn drove antibody-dependent tumor cell phagocytosis and effective presentation to evoke cytotoxic CD8^+^ T cell responses, shaping the TME to boost immune machinery for tumor cell killing. At the clinical end, the adoptive transfer of variant-harboring memory B cells facilitated tumorigenesis suppression. Collectively, our study provides insightful clues about the potential clinical benefits of a germline coding variant in human IgG1 and the manipulation of IgG1^+^ B cells in tumor immunotherapy.

## Results

### Human IgG1-G396R is a protective variant for CRC.

**Figure 1:**
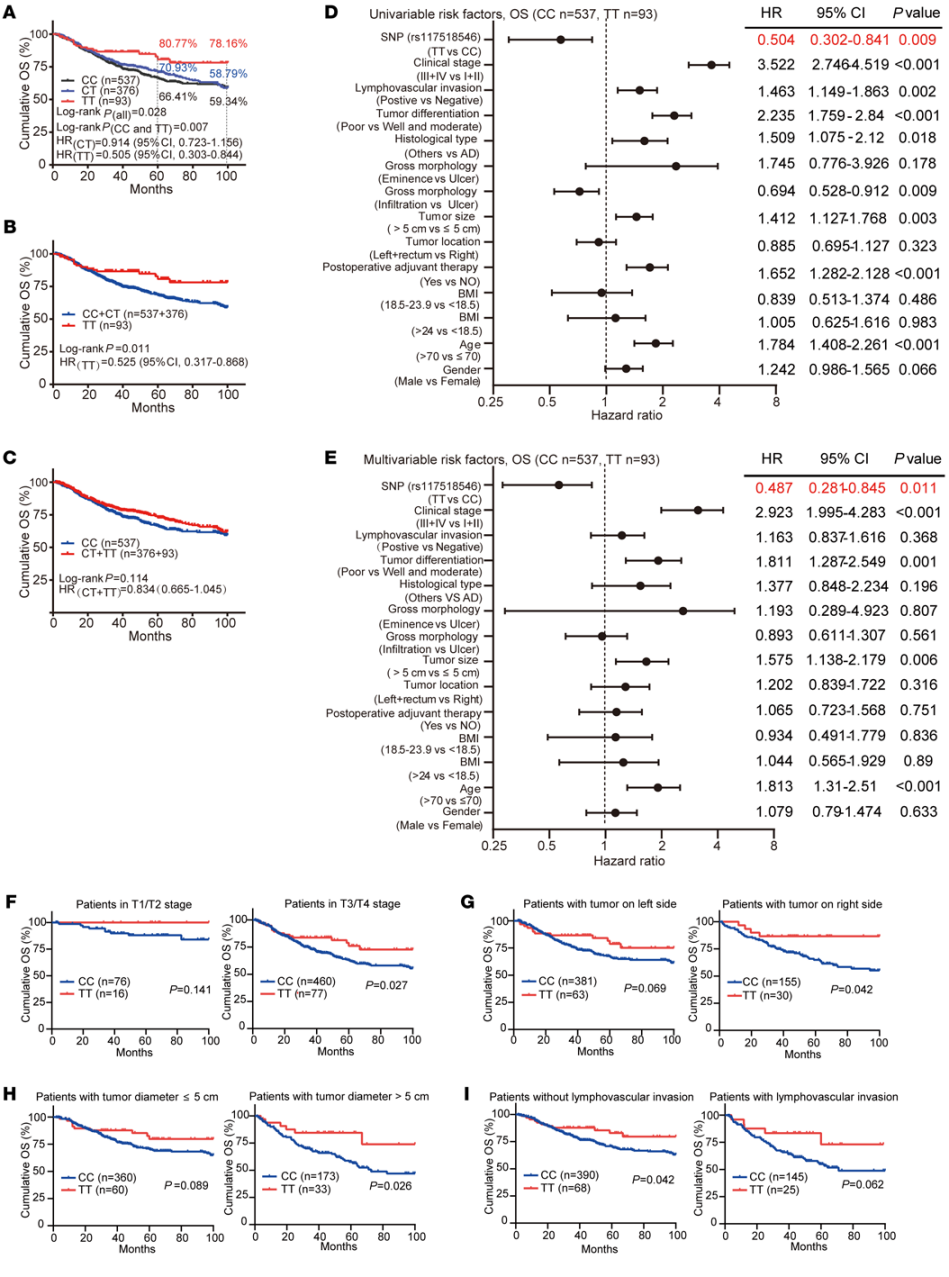
The hIgG1-G396R variant protects against colorectal tumorigenesis and progression. (**A**) Survival analyses of 1006 CRC patients with available follow-up information stratified by genotype. WT is indicated as the CC genotype, hIgG1-G396R heterozygous is denoted as CT, and hIgG1-G396R homozygous is denoted as TT. (**B** and **C**) Survival curves for 1006 CRC patients, classified by (**B**) recessive model and (**C**) dominant model. (**D** and **E**) Forest plots showing hazard ratios by (**D**) univariable COX regression and (**E**) multivariable COX regression analysis for correlation with the OS of CRC patients. (**F**–**I**) OS curves of 1006 CRC patients with different clinical treatments and clinical manifestations. Statistical significance was determined using a log-rank test (**A**–**C** and **F**–**I**). HR, hazard ratio; CI, confidence interval. Data are presented as the mean ± SEM.

Since the hIgG1-G396R variant has a strong Asian population specificity (Supplemental Figure 1, A and B; supplemental material available online with this article; https://doi.org/10.1172/JCI153454DS1), we thus analyzed a cohort containing 1006 CRC patients with 100-month survival clinical records from Peking University, People’s Hospital in Beijing, China, to investigate the correlation of the hIgG1-G396R variant with clinical colorectal tumorigenesis (Supplemental Table 1 and Supplemental Figure 1, C and D). The frequency of the hIgG1-G396R homozygotes was mildly decreased in the CRC patients compared with criteria-matched controls (1006 CRC patients versus 583 healthy controls, odds ratio = 0.747, *P* = 0.049; Supplemental Table 2), suggesting a potential protective role of this variant in CRC. As an in-depth validation, we compared the cumulative overall survival (OS) of all these 537 wild-type (WT), 376 hIgG1-G396R heterozygous, and 93 hIgG1-G396R homozygous CRC patients, and found that hIgG1-G396R homozygous patients exhibited substantially improved OS compared with both hIgG1-G396R heterozygous and WT patients (log-rank *P* = 0.028 under additive model, Figure 1A; log-rank *P* = 0.011 under recessive model, Figure 1B; log-rank *P* = 0.114 under dominant model, Figure 1C). Remarkably, the proportion of 100-month cumulative OS was 78.16% (95%CI, 68.59%–89.06%) for hIgG1-G396R homozygous patients, compared with 59.34% (95%CI, 54.29%–64.85%) for WT patients and 58.79% (95%CI, 52.61%–65.68%) for hIgG1-G396R heterozygous patients. And the proportions of 5-year cumulative OS were 66.41% (95%CI, 62.12%–71%) for WT patients, 77.93% (95%CI, 66.14%–76.06%) for hIgG1-G396R heterozygous patients, and 80.77% (95%CI, 72.09%–90.49%) for hIgG1-G396R homozygous patients. Notably, multivariable COX regression analyses implicated the hIgG1-G396R variant as an independent positive prognostic factor for OS (Figure 1, D and E, and Supplemental Table 3). Furthermore, hIgG1-G396R was consistently identified as a protective factor in OS (Figure 1, F–I, and Supplemental Figure 2) among CRC patients with different treatments and clinical manifestations.

Moreover, the hIgG1-G396R variant was also significantly associated with superior progression-free survival (PFS) in a large proportion of CRC patients (*n =* 966), which had obtainable follow-up PFS clinical information from the original 1006 patients. Consistent with the OS analysis, hIgG1-G396R again improved the PFS of CRC patients (log-rank *P* = 0.052 under additive model, Supplemental Figure 3A; log-rank *P* = 0.027 under recessive model, Supplemental Figure 3B; log-rank *P* = 0.089 under dominant model, Supplemental Figure 3C). The proportion of 100-month cumulative PFS was 75.75% (95%CI, 66.51%–86.27%) for hIgG1-G396R homozygous patients, compared with 58.47% (95%CI, 52.41%–65.24%) for hIgG1-G396R heterozygous patients and 57.87% (95%CI, 52.92%–63.29%) for WT patients. And the proportions of 5-year cumulative PFS were 62.92% (95%CI, 58.54%–67.62%) for WT patients, 66.89% (95%CI, 61.90%–72.28%) for hIgG1-G396R heterozygous patients, and 75.75% (95%CI, 66.51%–86.27%) for hIgG1-G396R homozygous patients. Moreover, multivariable COX regression analyses (Supplemental Figure 3, D and E, and Supplemental Table 3), survival analyses based on different characteristics (Supplemental Figure 3F), and allele frequency–based stratification analyses (Supplemental Table 4) implicated the hIgG1-G396R variant as an independent positive prognostic factor.

Because microsatellite instability (MSI) status is important for TAA load and immune responses in CRC (19), we carried out survival curve analysis and multivariable regression analysis on 292 patients with available information for either MSI or microsatellite stability (MSS) clinical data. Clearly, hIgG1-G396R showed protection in both the MSI and MSS groups (Supplemental Figure 2E, Supplemental Figure 3F, and Supplemental Figure 4). It is worth noting that hIgG1-G396R confers greater benefits (CC vs. TT genotype) in MSS patients compared with MSI patients. Thus, hIgG1-G396R is a protective germline variant for progression and survival in CRC patients.

### Murine homolog of hIgG1-G396R protects mice from tumorigenesis.

**Figure 2:**
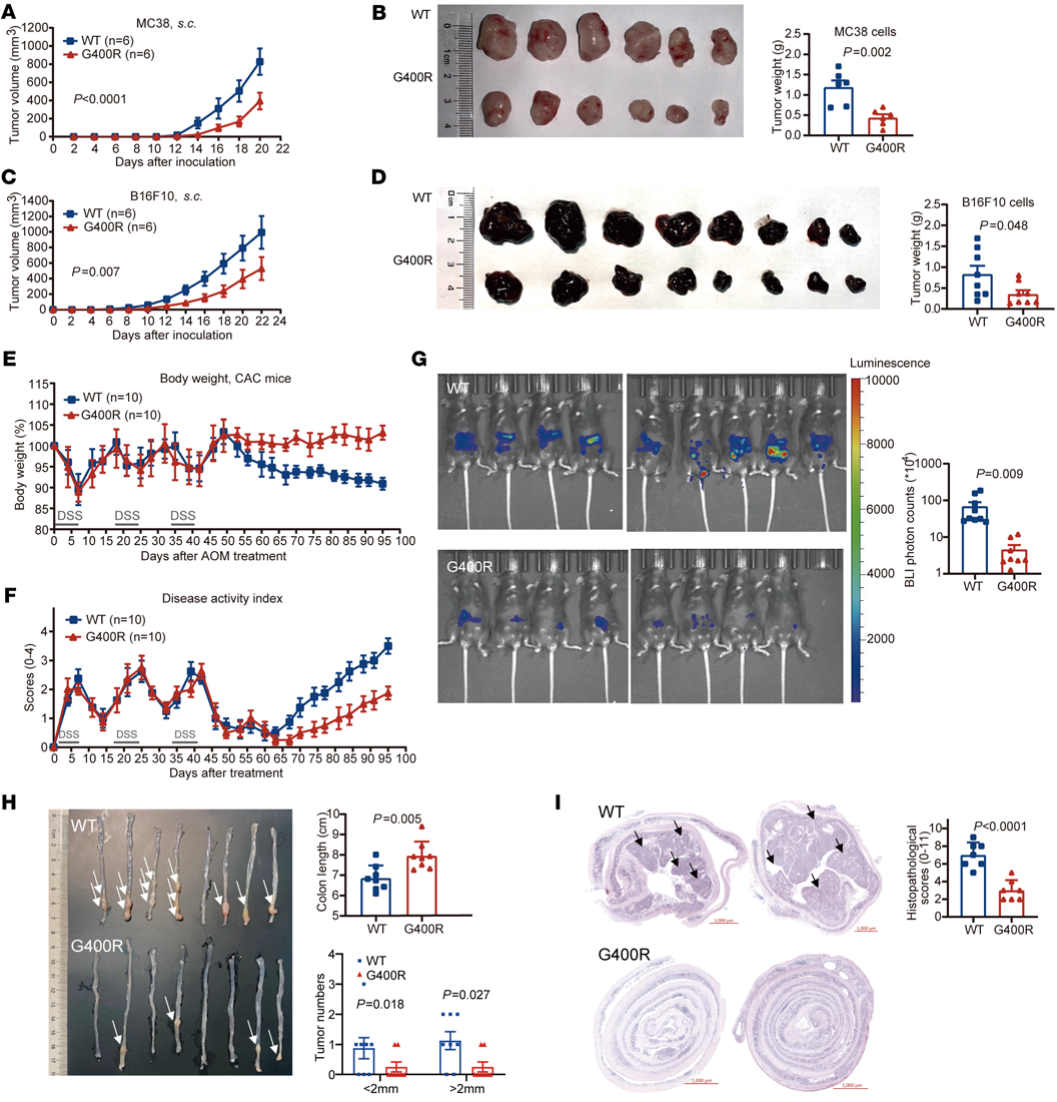
The murine homolog mIgG2c-G400R variant enhances tumor resistance in mice. (**A**) MC38 tumor growth and (**B**) tumor weight at the time of sacrifice of WT and mIgG2c-G400R mice. (**C**) B16F10 tumor growth and (**D**) tumor weight of WT and mIgG2c-G400R mice. (**E**) Body weights and (**F**) disease activity index of AOM/DSS-induced CAC model for both WT and mIgG2c-G400R mice. (**G**) Bioluminescence images with injection of L-012 solution on week 16 after AOM injection. (**H**) Representative images and quantification of colon tumor numbers in mice of indicated genotypes. Tumors are indicated by white arrows. (**I**) Representative photographs of H&E-stained colon cross sections and histopathological scores after termination of the experiment. The black arrows indicate the tumors in the colon. Scale bars: 1000 μm. One of 3 representative experiments is shown. Statistical significance was determined using 2-way ANOVA (**A** and **C**) or an unpaired, 2-tailed Student’s *t* test (**B**, **D**, **G**, **H**, and **I**). HR, hazard ratio. CI, confidence interval. Data are presented as the mean ± SEM.

To determine the mechanism of action of hIgG1-G396R in inhibiting CRC tumorigenesis and progression, we first utilized the previously constructed mIgG1-G390R–knockin mice (17) and applied the syngeneic MC38 murine colon carcinoma model and B16F10-mOVA transplantation tumor model. Clearly, mIgG1-G390R did not show obviously protective effects against tumors in terms of tumor growth inhibition and improved host mouse survival compared with WT (Supplemental Figure 5, A and B). This observation is consistent with documented conclusions that murine IgG1 has relatively low affinity for murine activating FcγRs, which are important for antibody-derived antitumor effects, including ADCP and ADCC, in establishing effective antitumor immunity (20–22). It binds more avidly to murine inhibitory FcγRIIB than to murine activating FcγRs, and fails to activate complement by the classical pathway, resembling human IgG4 in that it had limited ability to induce effector functions, which represents a marked difference from human IgG1 (23, 24). In consideration of murine IgG2c sharing a conserved tail sequence and functional equivalence to human IgG1 in terms of similar binding affinity for activating versus inhibitory FcγRs when considering the Fc portion–mediated effects of antibodies (25), we therefore generated knockin mice harboring the homologous mutation at the cytoplasmic tail of endogenous murine IgG2c (denoted mIgG2c-G400R; Supplemental Figure 5, C and D). In a transplantation tumor model using MC38 cells, which presents a broad TAA pool (26), mIgG2c-G400R mice showed significantly slower progression of inoculated MC38 tumor cells compared with the WT control mice (Figure 2, A and B). As an extension, we also applied the B16F10 autologous transplantation tumor model, which has relatively lower tumor mutational load (27), and found that mIgG2c-G400R mice also showed decreased B16F10 tumor progression compared with WT mice (Figure 2, C and D). In order to more closely simulate colon tumorigenesis in humans, we introduced the azoxymethane/dextran sodium sulfate (AOM/DSS) colitis-associated carcinoma (CAC) model to our study, which has been widely used for the induction of colitis-dependent neoplasia and primary cancer in mice (Supplemental Figure 5E and refs. 28, 29). Upon AOM/DSS treatment, the mIgG2c-G400R mice exhibited reduced weight loss (Figure 2E), decreased disease activity index (Figure 2F), alleviated phenotypes of tumor-associated inflammation (Figure 2G and Supplemental Figure 5, F and H), reduced tumor burden (Figure 2H and Supplemental Figure 5G), and ameliorated histopathological changes (Figure 2I and Supplemental Figure 5I) compared with the WT control mice. Thus, the mIgG2c-G400R variant alleviates tumorigenesis and tumor progression in a murine tumor model, recapitulating the results of the functionally equivalent hIgG1-G396R variant in human CRC patients.

### hIgG1-G396R promotes plasma cell infiltration in the TME.

**Figure 3:**
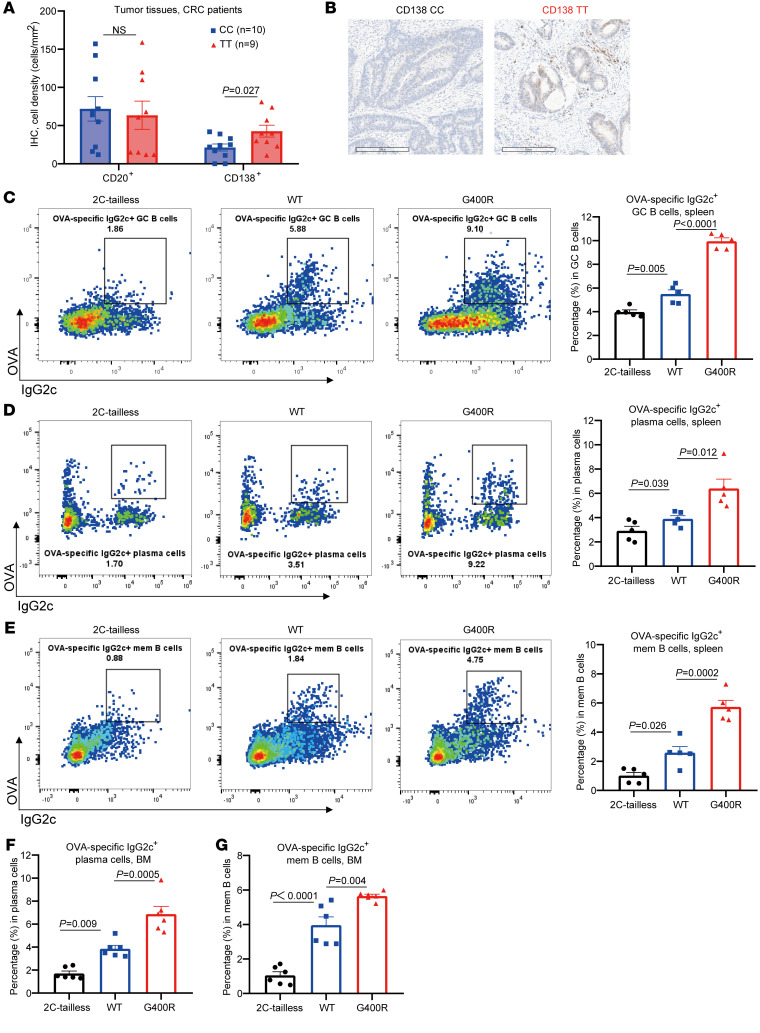
The hIgG1-G396R variant promotes plasma cell differentiation. (**A**) The absolute numbers of B cells (CD20^+^) and plasma cells (CD138^+^) in tumor tissues from CRC patients detected by IHC. (**B**) Representative microphotographs of CD138 IHC staining in tumor specimens from CRC patients. Scale bars: 200 μm. (**C**–**G**) Flow cytometry analyses to assess the relative percentages of (**C**) OVA-specific IgG2c^+^ GC B cells, (**D**) plasma cells, (**E**) memory B cells in the spleen, (**F**) plasma cells, and (**G**) memory B cells in the bone marrow from mIgG2c-tailless, WT, and mIgG2c-G400R mice at week 2 after recall immunization. One of 3 representative experiments is shown. Statistical significance was determined using an unpaired, 2-tailed Student’s *t* test (**A**) or 1-way ANOVA (**C**–**G**). Data are presented as the mean ± SEM. NS, not significant.

To reveal the mechanism of how hIgG1-G396R alleviates tumorigenesis and tumor progression in CRC patients, we first examined B cell subpopulations in the CRC TME. There were elevated numbers of CD138^+^ plasma cells in the tumor specimens of hIgG1-G396R homologous CRC patients compared with those of WT patients based on the immunohistochemistry (IHC) results, although there were comparable numbers of total B cells (Figure 3, A and B). Similarly, higher percentages of class-switched IgG2c^+^ plasma cells were observed in the murine mesenteric lymph nodes (mLNs), the colonic lamina propria (LP) of AOM/DSS-induced mIgG2c-G400R CAC mice (Supplemental Figure 6, A–F), and the tumor-draining LNs (TDLNs) of MC38 tumor–bearing mIgG2c-G400R mice (Supplemental Figure 6, G–I). These results suggest hIgG1-G396R and its murine homolog enhance plasma cell differentiation in the TME. As a more vigorous validation, we performed a standard ovalbumin (OVA) model antigen–based immunization experiment in WT, mIgG2c-G400R, and an additionally constructed strain of mIgG2c-tailless mice with a truncated cytoplasmic tail of the membrane-bound mIgG2c heavy chain, which blocks the mIgG-tail signaling–induced enhancement of IgG2c^+^ B cell activation and differentiation (30, 31). Consistently, we detected an increase in OVA-specific IgG2c^+^ plasma cells and memory B cells, as well as higher percentages of germinal center (GC) B cells in the spleen of mIgG2c-G400R mice compared with WT mice (Figure 3, C–E). Moreover, the substantial increases in OVA-specific IgG2c^+^ plasma cells and memory B cells in mIgG2c-G400R mice were also detected in the bone marrow (Figure 3, F and G). Thus, it is clear that hIgG1-G396R promotes class-switched memory B cell and plasma cell differentiation in the TME.

### hIgG1-G396R shapes the TME with enhanced infiltration of cytotoxic CD8^+^ T cells and DCs.

**Figure 4:**
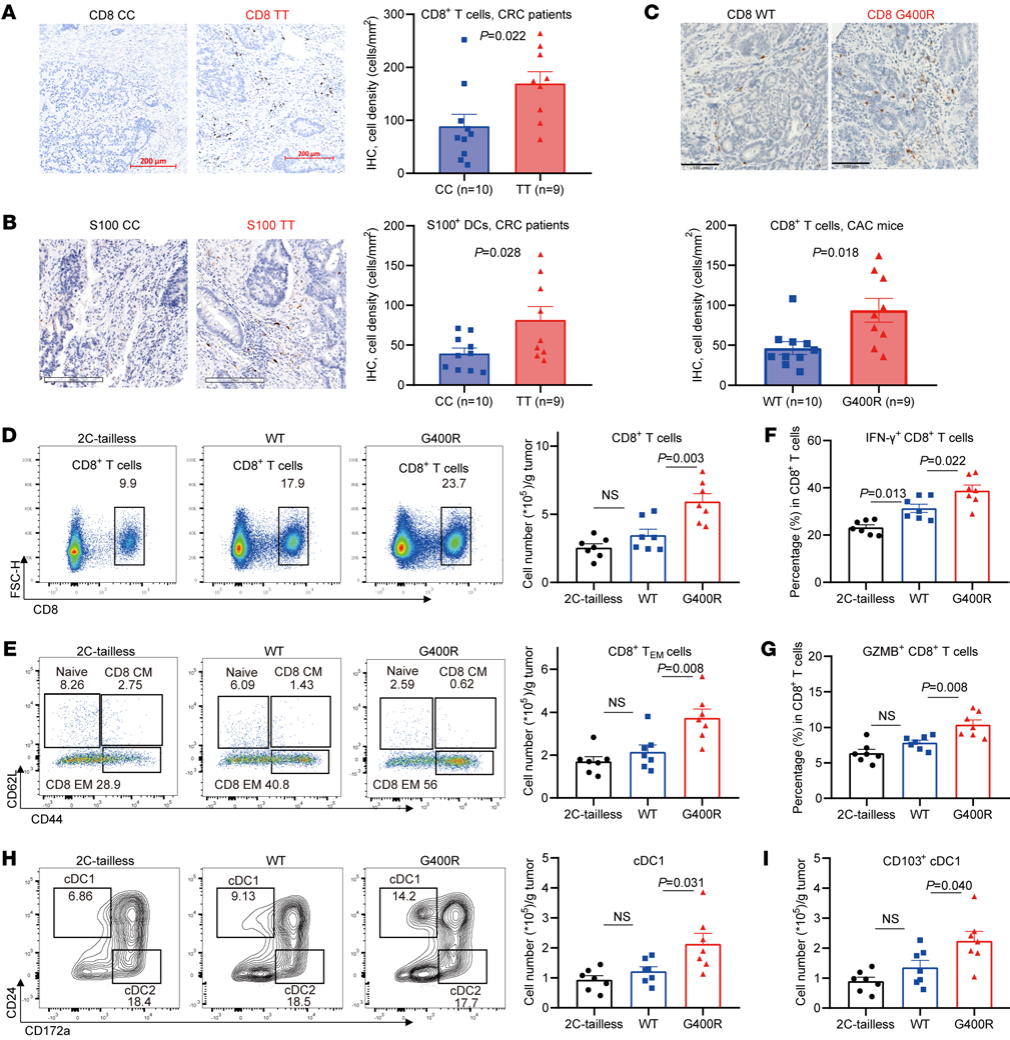
Elevated numbers of infiltrating CD8^+^ T cells and DCs in the TME of the hIgG1-G396R variant. (**A** and **B**) Representative micrographs and cell density (in mm^2^) of (**A**) CD8^+^ T cells and (**B**) S100^+^ DCs in tumor sections from hIgG1-G396R homozygous and WT CRC patients by IHC. Scale bars: 200 μm. (**C**) Representative micrographs and cell density (in mm^2^) of CD8^+^ T cells in colon specimens from CAC-induced WT and mIgG2c-G400R mice, measured by IHC. Scale bars: 100 μm. (**D**) Quantification by flow cytometry, pregated on live CD45^+^ cells, of tumor-infiltrating CD8^+^ T cells in the tumor tissues on day 10 after MC38-mOVA tumor cell inoculation. (**E**) Quantification of tumor-infiltrating naive, central memory, and effector memory CD8^+^ T cells in the MC38-mOVA tumor tissues by flow cytometry, pregated on CD45^+^, CD3^+^, and CD8^+^ T cells. (**F** and **G**) Quantification of (**F**) IFN-γ–secreting and (**G**) granzyme B–secreting (GZMB-secreting) CD8^+^ T cells, pregated on CD45^+^, CD3^+^, and CD8^+^ T cells. (**H**–**I**) Quantification of tumor-infiltrating DC subtypes. One of 3 representative experiments is shown (**D**–**I**). Statistical significance was determined using an unpaired, 2-tailed Student’s *t* test (**A**–**C**) or 1-way ANOVA (**D**–**I**). Data are presented as the mean ± SEM.

Because the interactions between the immune system and tumors are governed by a complex network of cell-cell interactions, we proposed that the hIgG1-G396R variant may exert a pleiotropic immunomodulatory role by also affecting interrelated immune cells for effective immunosurveillance. To test this hypothesis, we analyzed the effect of this variant on infiltration of both innate and adaptive immune cells in the TME of CRC patients, including T cells, natural killer (NK) cells, macrophages, and DCs (Figure 4, A and B, and Supplemental Figure 7A). We found that hIgG1-G396R homozygotes had prominently increased tumor-infiltrating CD8^+^ T cells (Figure 4A) and S100^+^ DCs (Figure 4B) compared with WT patients. Single-cell RNA sequencing (scRNA-seq) is widely used to profile immune cells in organs and tissues. Thus, we reanalyzed the proportions of immune cell subsets in the TME of 18 CRC patients using the scRNA-seq results from our recent studies (Supplemental Figure 7, B and C, and refs. 32, 33). Both patient genome sequencing and single B cell RNA-seq data identified 11 heterozygotes, 6 WT, and only 1 hIgG1-G396R homozygote within these 18 CRC patients, so we could only compare immune cell profiles within the TME from 12 hIgG1-G396R carriers (CT and TT) to those from 6 noncarriers (CC) (Supplemental Table 5). Nevertheless, in hIgG1-G396R carriers, there were increased proportions of *IgG*^+^ plasma cells, *CXCR5*^+^ T follicular helper cells, *CD6*^+^ tumor-resident memory T cells, and decreased proportions of *LAYN*^+^ exhausted T cells (Supplemental Figure 7D), implying that the variant favors the formation of an antitumor microenvironment.

We next examined the tumor-infiltrating immune cells in CAC mice. IHC revealed that the colon tumor specimens from CAC-induced mIgG2c-G400R mice exhibited increased infiltration of CD8^+^ T cells compared with those from WT mice (Figure 4C), while they exhibited no significant differences in CD4^+^ T cells, NK cells, and macrophages (Supplemental Figure 7E). We also found that the infiltration of CD8^+^ T cells and IFN-γ^+^ CD8^+^ T cells in the LP and mLNs was slightly increased in mIgG2c-G400R mice (Supplemental Figure 7, F–H). In the MC38 tumor model, mIgG2c-G400R mice also exhibited increased amounts of CD8^+^ T cells within subcutaneous tumor tissues (Figure 4D), and especially increased CD44^hi^CD62L^lo^CD8^+^ effector T cells (Figure 4E), with elevated levels of cytolytic IFN-γ (Figure 4F) and granzyme B production (Figure 4G), suggesting that tumor-infiltrating CD8^+^ T cells from MC38-inoculated mIgG2C-G400R mice maintain an optimal functionality that may help to limit tumor progression. Notably, DCs, especially CD103^+^ type I conventional DCs (cDC1s), which are the key antigen-presenting cells that transport antigens to lymphoid structures and prime tumor-specific CD8^+^ T cells (34), were also elevated in mIgG2c-G400R mice compared with WT mice (Figure 4, H and I). The numbers of tumor-infiltrating macrophages and NK cells displayed no significant differences (Supplemental Figure 7I).

As we observed elevated CD8^+^ T cell infiltration and activation in hIgG1-G396R homozygotes and CD8^+^ T cells are one of the key tumor cytotoxic effector cells in antitumor immunity, we therefore supposed that CD8^+^ T cells might be essential for protective effects in antitumor responses conferred by hIgG1-G396R. Thus, we applied CD8^+^ T cell depletion in WT and mIgG2c-G400R mice by anti-CD8a antibody treatment before and during MC38 tumor model induction (Supplemental Figure 7J), and found that the enhanced antitumor effect of mIgG2c-G400R mice does depend on the normal function of CD8^+^ T cells to some extent (Supplemental Figure 7K). In addition, T cell adoptive transfer experiments verified this hypothesis (Supplemental Figure 7, L and M). Collectively, these data suggest that hIgG1-G396R stimulates the infiltration of cytotoxic CD8^+^ T cells and DCs to potentiate antitumor activity in the TME.

### hIgG1-G396R favors the formation of TLSs in tumor tissues.

**Figure 5:**
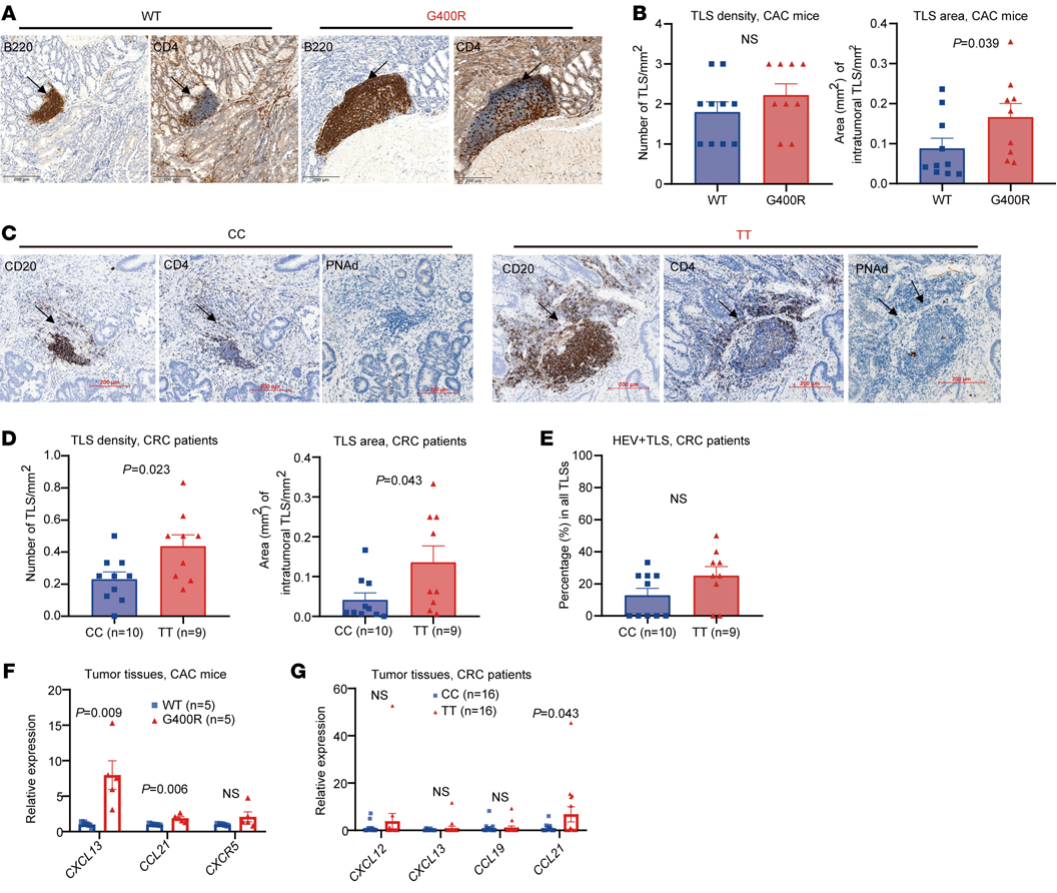
hIgG1-G396R promotes the formation of tertiary lymphoid structures in tumor tissues. (**A**) Microphotographs of representative tertiary lymphoid structures (TLSs) located in the tumor section of CAC-induced mice. Black arrows indicate TLSs. Scale bars: 200 μm. (**B**) IHC showing the numbers and relative areas of TLSs in CAC-induced mice. (**C**) Representative IHC of TLSs and PNAd^+^ TLSs within the tumor specimens from CRC patients, as indicated by black arrows. Scale bars: 200 μm. (**D** and **E**) The numbers of TLSs per mm^2^, relative TLS area, and the percentages of HEV^+^ TLSs for each genotype are shown. (**F**) The expression levels of several chemokine genes associated with TLS formation in the colon tumor tissues from CAC-induced mice by RT-qPCR assays. (**G**) The expression levels of several chemokine genes in the tumor specimens from CRC patients quantified by RT-qPCR assays. One of 3 representative experiments is shown (**F** and **G**). Statistical significance was determined using an unpaired, 2-tailed Student’s *t* test. Data are presented as the mean ± SEM.

TLSs were recently identified as important indicators of favorable outcomes of immunotherapies (9–11). B cells and DCs have been shown to be the major initiators of TLS formation, and together with T cells are key constituents of TLSs (35). IHC results in our experimental system revealed larger TLS formation within the colon tumors of mIgG2c-G400R CAC mice (Figure 5, A and B). Importantly, the elevated TLS formation was largely recapitulated in the tumor tissues of CRC patients, with obviously increased TLS density and TLS area in the tumor specimens from hIgG1-G396R homozygotes in comparison with WT (Figure 5, C and D). We also detected peripheral node addressin (PNAd) via IHC, a specific marker for high endothelial venules (HEVs) (36), and found increased proportions of HEV^+^ TLSs in the tumor specimens of hIgG1-G396R homozygous patients (Figure 5E), suggesting increased TLS formation and recruitment of lymphocytes in hIgG1-G396R homozygotes. The transcriptional levels of 5 chemokines (*CXCR5*, *CXCL12*, *CXCL13*, *CCL19*, and *CCL21*) that have been reported to be involved in the formation of TLSs (37) were also prominently elevated in both the tumor specimens from mIgG2c-G400R mice (Figure 5F) and hIgG1-G396R homozygous CRC patients (Figure 5G). Collectively, these findings suggest that the hIgG1-G396R variant might potentiate antitumor responses also by invigorating CD8^+^ effector T cell priming and activation within TLSs with enhanced activity. Although the T cell and B cell interaction in the TME is important for T cell effector function in antitumor immunity, we detected no significant difference in the major histocompatibility complex (MHC) and costimulatory molecules CD86 on the cell surface of mIgG2c-G400R versus WT IgG2c^+^ B cells in the TME (Supplemental Figure 8A), suggesting that the mIgG2c-G400R variant does not seem to drastically alter the priming function of B cells to activate T cells.

### hIgG1-G396R induces a burst of tumor-specific IgG antibody production.

**Figure 6:**
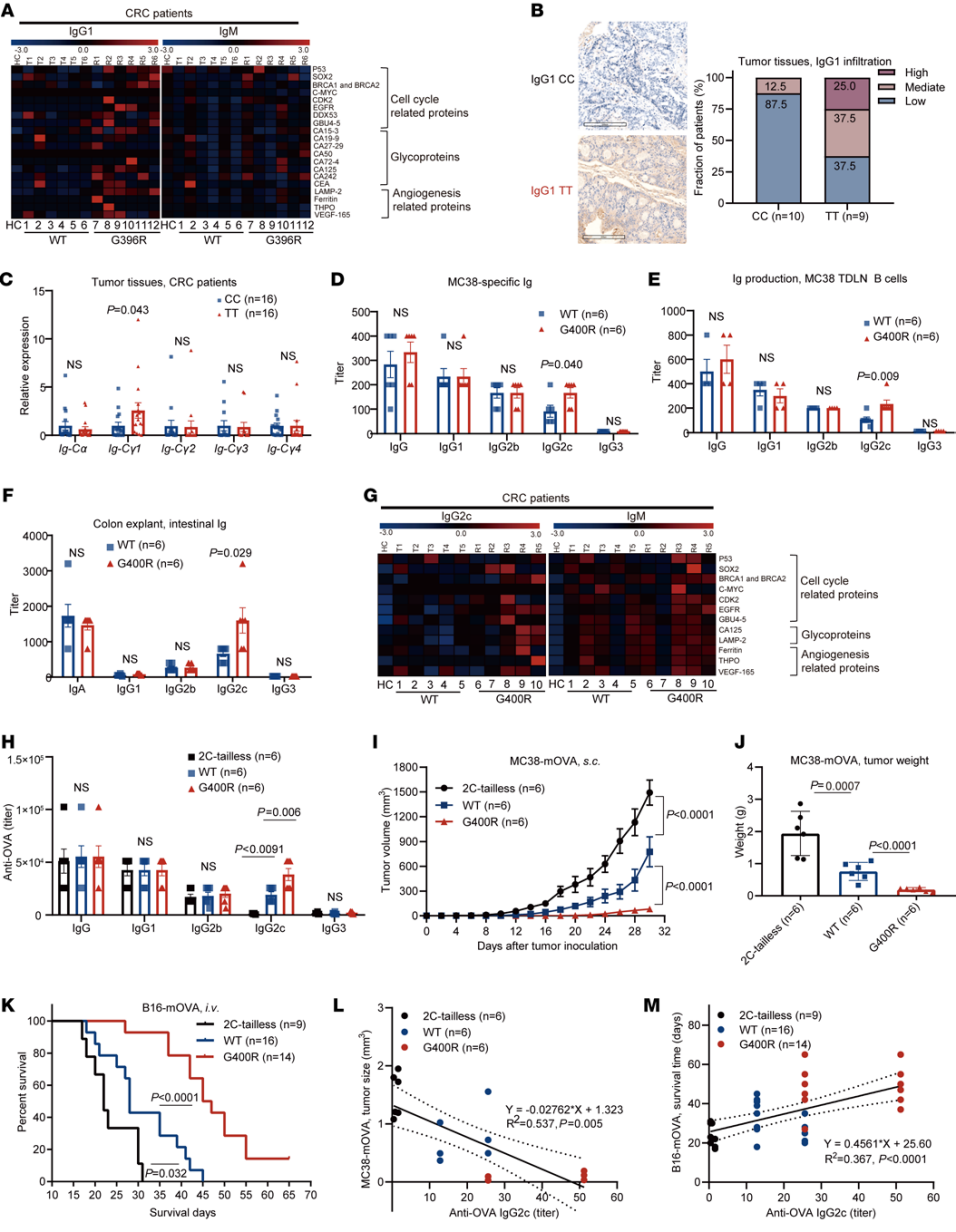
The hIgG1-G396R variant potentiates tumor-specific IgG antibody production. (**A**) Heatmap showing the detection of TAA- and autoantigen-specific IgG1 and IgM in the plasma samples of 12 CRC patients and 1 healthy donor. (**B**) Representative microphotographs of IgG1 IHC in tumor specimens from CRC patients. Scale bars: 200 μm. (**C**) The transcriptional levels of germline immunoglobulin α-chain constant region (*Cα*) and γ-chain constant region 1 (*C*γ*1*), *C*γ*2*, *C*γ*3*, and *C*γ*4* in the tumor tissues of CRC patients, measured by RT-qPCR analyses. (**D**) Detection of MC38-specific IgG and IgG subclasses by ELISA in serum on day 20 after tumor inoculation. (**E**) Detection of IgG subclass secretion in supernatants of in vitro–activated MC38 TDLN B cells. (**F**) ELISA quantification of IgG subclasses and IgA in supernatants of colon explants isolated from CAC-bearing mice. (**G**) Heatmap showing the levels of IgG2c and IgM, which are specific for TAAs and autoantigens, in the serum samples of CAC-induced mice. (**H**) Detection of anti-OVA antibodies in serum from mIgG2c-tailless, WT, and mIgG2c-G400R mice on week 2 after recall immunization with OVA. (**I**) Tumor growth and (**J**) tumor weight of MC38-mOVA cells in OVA-immunized mIgG2c-tailless, WT, and mIgG2c-G400R mice. (**K**) Survival curves of OVA-immunized mice after intravenous injection of B16-mOVA tumor cells. (**L**) Linear regression analyses between the titer of OVA-specific IgG2c and MC38-mOVA tumor size, as indicated by solid lines. Dotted lines indicate 95%CI. (**M**) Linear regression analyses between the titer of OVA-specific IgG2c and survival day after intravenous injection of B16-mOVA tumor cells, as indicated by solid lines. Dotted lines indicate 95%CI. One of 3 representative experiments is shown (**C**–**F** and **H**–**M**). Statistical significance was determined using an unpaired, 2-tailed Student’s *t* test (**B**–**F**), 1-way ANOVA (**H** and **J**), 2-way ANOVA (**I**), log-rank test (**K**), or linear regression (**L** and **M**). Data are presented as the mean ± SEM. NS, not significant.

Given that the hIgG1-G396R variant enhances IgG1^+^ B cell activation and IgG1-producing plasma cell differentiation (17), and that human IgG1 antibodies are important modulators in immunosurveillance (38, 39), we investigated whether the hIgG1-G396R variant differentially regulates the production of tumor-specific IgG1 antibodies. To comprehensively characterize the TAA-specific antibodies, we used microarrays to profile tumor-specific antibody levels against a panel of TAAs and autoantigens. In CRC patients, the hIgG1-G396R homozygotes produced elevated levels of TAA-specific IgG1 antibodies, while this stimulatory effect was not significant for IgM antibodies (Figure 6A and Supplemental Figure 8B). More importantly, elevated infiltration of IgG1 (Figure 6B) in tumor sections from hIgG1-G396R homozygous CRC patients was consistently detected by IHC. Specifically, we detected higher levels of immunoglobulin heavy constant γ 1 (*C*γ*1*) transcripts in the tumor tissues from hIgG1-G396R homozygous CRC patients, which is a hallmark of elevated IgG1 expression in the TME (40), while the levels of *C*γ*2*, *C*γ*3*, *C*γ*4*, and immunoglobulin heavy constant α (*C*α) transcripts were comparable (Figure 6C).

In the MC38 transplantation model, mIgG2c-G400R mice generated elevated amounts of MC38 cell–specific IgG2c antibodies compared with WT mice (Figure 6D). Tumor-primed B cells isolated from the TDLNs of mice could secret large amounts of IgG under the in vitro restimulation with tumor cells, compared with unprimed B cells (Supplemental Figure 8C and ref. 41). B cells from the TDLNs of mIgG2c-G400R mice secreted significantly higher amounts of IgG2c upon restimulation with irradiated MC38 cells than B cells from TDLNs of WT mice (Figure 6E). Consistently, the levels of intestinal IgG2c antibodies were significantly increased in the colon explants of mIgG2c-G400R mice upon AOM/DSS induction, while the levels of intestinal IgA, IgG1, IgG2b, and IgG3 antibodies in mIgG2c-G400R mice were comparable to WT mice (Figure 6F). Sera TAA-specific antibody analyses further validated the burst of IgG2c antibody production, but not IgM, IgG1, or IgG2b antibodies in mIgG2c-G400R CAC mice (Figure 6G and Supplemental Figure 8D). And the boosted production of TAA-specific IgG2c antibody was also detected in the colon explants of CAC-induced mice by TAA microarrays (Supplemental Figure 8E). Together, these results demonstrate that the hIgG1-G396R variant boosts the production of TAA-specific IgG1 antibodies in human CRC patients, similar to the mIgG2c-G400R variant in tumor-bearing mice.

To investigate the role of this variant in immunosurveillance mediated by TAA-specific IgG2c antibody upregulation, we utilized membrane-bound-OVA-expressing MC38 tumor cells (denoted MC38-mOVA) and B16F10-mOVA (denoted B16-mOVA) (Supplemental Figure 8, F and G) to examine the effect of OVA priming on antitumor immunity in the mIgG2c-G400R mice. To establish a loss-of-function control in our experimental system, we again included the mIgG2c-tailless mouse in this study that has blunted mIgG-tail signaling (30, 31). After immunization of OVA antigen combined with Th1-prone adjuvant (42), which favors IgG2c antibody production, OVA-specific IgG2c antibody production increased in mIgG2c-G400R mice and decreased in mIgG2c-tailless mice compared with WT mice (Figure 6H). Notably, tumor growth was clearly inhibited in mIgG2c-G400R mice during the subsequent MC38-mOVA challenge, whereas it was exacerbated in mIgG2c-tailless mice compared with WT mice (Figure 6, I and J). Consistently, in the B16-mOVA tumor cell intravenous transplantation model, mIgG2c-G400R mice exhibited prolonged survival compared with WT mice, while the benefits were diminished in mIgG2c-tailless mice (Figure 6K). Further linear regression fitting analyses indicated that OVA-specific IgG2c antibody levels negatively correlated with MC38-mOVA tumor size (Figure 6L) and positively correlated with survival time in the B16-mOVA transplantation tumor model (Figure 6M). Thus, elevated TAA-specific IgG2c antibody caused by the mIgG2c-G400R variant strengthens immunosurveillance of tumors.

### hIgG1-G396R potentiates ADCP and antigen presentation in antitumor immunity.

**Figure 7:**
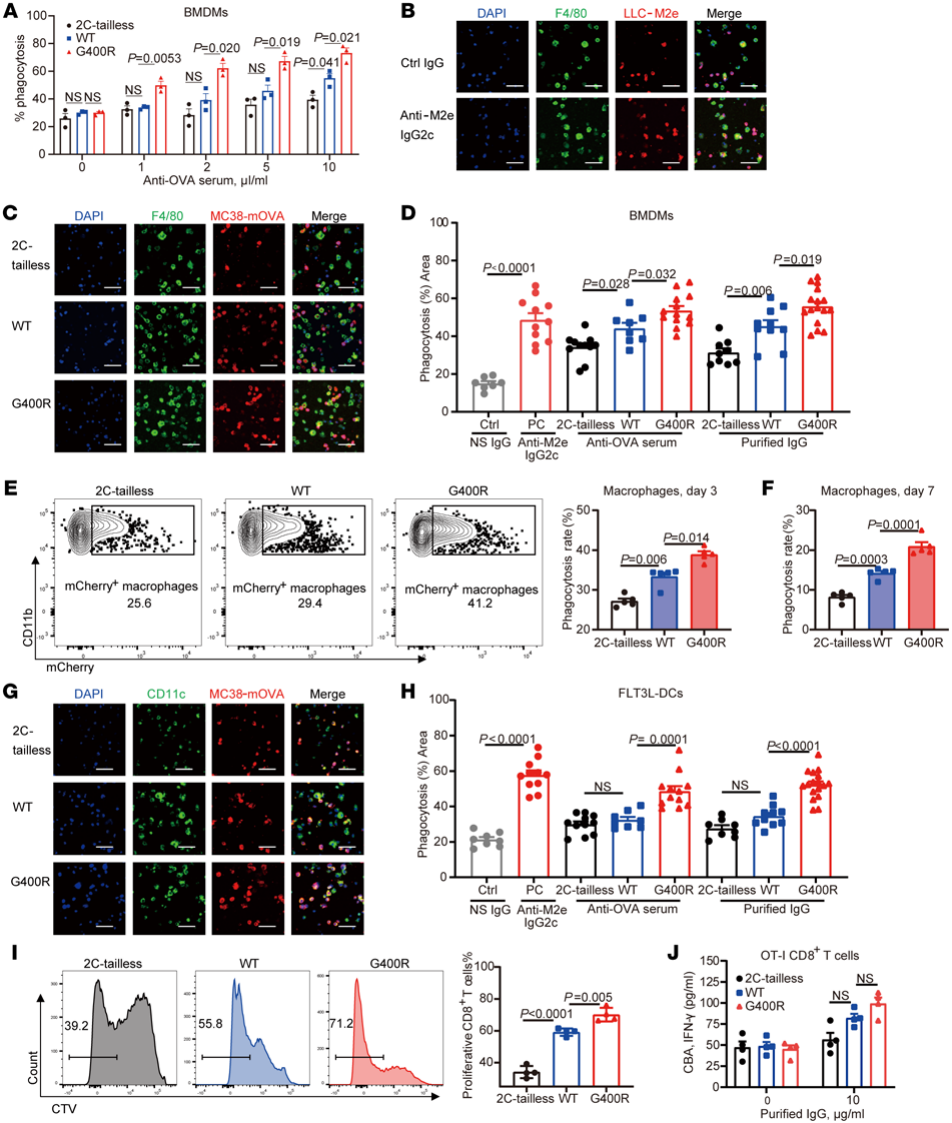
Enhanced ADCP activity and CD8^+^ T cell priming are mediated by tumor-specific antibodies. (**A**) Comparison of ADCP activities of necrotic tumor cells by BMDMs using serum samples from OVA-immunized mIgG2c-tailless, WT, and mIgG2c-G400R mice, detected by flow cytometry. (**B** and **C**) Assessment of the ADCP efficiency of BMDMs after coculture by fluorescence staining. Nuclei marked by DAPI staining (blue), macrophages labeled with F4/80-FITC (green), and engulfed tumor cells labeled with mCherry (red). Scale bars: 50 μm. (**D**) Percentage phagocytosis of tumor cells by BMDMs was analyzed based on the results from **B** and **C**. (**E** and **F**) Measurement of phagocytosis rates in vivo by MC38-mOVA tumor-infiltrating macrophages on (**E**) day 3 and (**F**) day 7. Phagocytizing macrophages were defined as CD11b^+^F4/80^+^mCherry^+^ cells and were measured with flow cytometry. (**G**) Representative confocal fluorescence images showing the process of phagocytosis mediated by FLT3L-DCs in the presence of tumor cell–specific antibody. Nuclei marked by DAPI staining (blue), DCs stained with CD11c-FITC (green), and tumor cells labeled with mCherry (red). Scale bars: 50 μm. (**H**) The percentages of mCherry^+^ DCs were recorded and used to calculate the levels of phagocytosis. (**I**) Quantification of CTV-labeled OT-I CD8^+^ T cells that had been cocultured with antibody-coated tumor cell–primed DCs. CD8^+^ T cells with low CTV staining indicate proliferating OT-I CD8^+^ T cells. (**J**) Production of IFN-γ by OT-I CD8^+^ T cells after coculturing with tumor-primed FLT3L-DCs, detected by IFN-γ cytometric bead array (CBA). One of 3 representative experiments is shown. Statistical significance was determined using 1-way ANOVA. Data are presented as the mean ± SEM.

Human IgG1 antibody, or the functionally equivalent murine IgG2c, can enhance antitumor activity via ADCP and ADCC (21, 22). To determine whether this mechanism is also triggered by the upregulation of anti-TAA antibodies in the carriers of this variant, we coincubated necrotic exogenous antigen–expressing tumor cells, consisting of either LLC-M2e (LLC cells expressing the influenza virus M2 ectodomain in 3 tandem repeats) or MC38-mOVA, with phagocytes differentiated in vitro in the presence of either anti-M2e IgG2c antibodies or OVA antiserum. We then calculated the efficiency of phagocytosis by flow cytometry and confocal fluorescence imaging. Indeed, anti-M2e IgG2c antibodies induced efficient phagocytosis of LLC-M2e cells by bone marrow–derived macrophages (BMDMs) in a dose-dependent manner (Supplemental Figure 8, H and I). More importantly, OVA antiserum or further purified IgG from OVA-immunized mIgG2c-G400R mice with MC38-mOVA induction showed more potent effects on BMDM activation and phagocytosis of antibody-targeted tumor cells (Figure 7A and Supplemental Figure 8J). The enhanced ADCP activity of BMDMs largely relied on dominant TAA-specific IgG2c antibodies in the serum, based on similar ADCP effects with purified IgG antibodies (Figure 7, B–D). As expected, tumor-infiltrating macrophages from MC38-mOVA–inoculated mIgG2c-G400R mice exhibited increased in vivo tumor-phagocytosing activity, compared with those from WT mice (Figure 7, E and F). Considering that tumor antigen presentation by DCs is crucial to initiate T cell–mediated protective immunity, we investigated the impact of mIgG2c-G400R on antibody-mediated tumor antigen uptake by FLT3L-DCs (43, 44). Similarly, FLT3L-DCs showed dose-dependent phagocytosis of antibody-coated tumor antigens examined by flow cytometry (Supplemental Figure 8K) and confocal fluorescence microscopy (Supplemental Figure 8L). We found that OVA antiserum and purified IgG antibodies from OVA-immunized mIgG2c-G400R mice enhanced the antigen uptake activities of FLT3L-DCs (Figure 7, G and H, and Supplemental Figure 8M), which in turn promoted OT-I CD8^+^ T cell activation and proliferation (Figure 7, I and J). Collectively, our results show that this variant strengthens antitumor effects in part by enhancing ADCP and subsequent antigen presentation.

### Enhanced antitumor efficiency by adoptive transfer of memory B cells harboring CRC protective variant.

**Figure 8:**
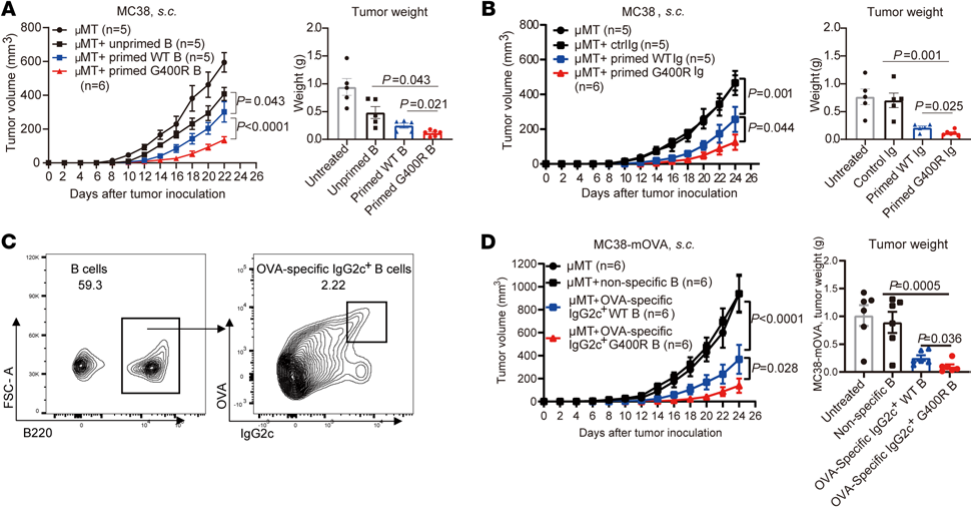
Tumor-specific antibodies and tumor-primed B cells from the mIgG2c-G400R mice contribute to higher antitumor efficiencies. (**A**) B cells purified from untreated mice, MC38 tumor–bearing WT, or mIgG2c-G400R mice were adoptively transferred into μMT mice followed by MC38 tumor inoculation. Tumor growth curve and tumor weight are shown. (**B**) IgG purified from the serum samples of untreated mice, MC38 tumor–inoculated WT, or mIgG2c-G400R mice were intravenously reinfused into μMT mice followed by MC38 tumor cell inoculation. Tumor growth was recorded and tumor weight is shown. (**C**) Representative flow cytometry histograms of OVA-specific IgG2c^+^ B cells isolated from OVA-immunized mice. (**D**) OVA-specific IgG2c^+^ B cells were adoptively transferred into μMT mice followed by MC38-mOVA tumor cell inoculation. The curves of MC38-mOVA tumor growth and tumor weights are shown. One of 3 representative experiments is shown. Statistical significance was determined using 2-way ANOVA (tumor volume in **A**, **B**, and **D**) or unpaired, 2-tailed Student’s *t* test (tumor weight in **A**, **B**, and **D**). Data are presented as the mean ± SEM.

The enhanced antitumor immunity in mIgG2c-G400R mice prompted us to assess whether adoptive transfer of tumor-specific memory B cells is a potential and feasible immunotherapy. First, we adoptively transferred MC38-primed B cells to B cell–deficient (μMT) mice followed by MC38 tumor inoculation. Clearly, tumor-primed B cells from mIgG2c-G400R mice conferred improved protection from tumor progression (Figure 8A). We next assessed the effects of tumor-specific IgG on antitumor response by IgG reinfusion to μMT mice followed by MC38 tumor inoculation. μMT mice with IgG reinfusion showed significantly decreased MC38 tumor progression compared with untreated μMT mice, and μMT mice showed much slower tumor growth after administration of IgG purified from MC38-inoculated mIgG2c-G400R mice (Figure 8B). Next, we transferred OVA-specific class-switched IgG2c^+^ B cells into μMT mice to detect the roles of TAA-specific class-switched B cells in antitumor immunity (Figure 8C), and found that OVA-specific IgG2c^+^ B cells from mIgG2c-G400R mice exhibited stronger antitumor effects (Figure 8D). These results demonstrate the potential therapeutic effect of tumor-primed highly reactive B cells and the feasibility of BCR manipulation in future immunotherapy.

## Discussion

Tumor-infiltrating B cells, including memory B cells and plasma cells, have been found in tumors and linked to antitumor activity in the TME (45). Although the impact of B cells on tumor progression is still somewhat controversial, depending on tumor types and stages (46, 47), emerging evidence supports the notion that the presence of tumor-infiltrating B cells in TLSs is an important prognostic indicator in different types of cancers (48, 49). With the perspective of a potential new hallmark for immunotherapy, our study here uncovers the roles of IgG1^+^ memory B cells in antitumor immunity in the context of CRC.

In this study, we identified an Asia-specific coding variant in human IgG1 that shows protective roles against colorectal tumorigenesis and progression. Although the mechanism of the population-specific distribution of this SNP (high minor allele frequency in East Asian population but low in other populations) is still elusive, perspectives from human evolution of immune-related genes could be a valuable clue. For example, introgressed Neanderthal and/or Denisovian alleles/loci are enriched for *HLA*, immunoglobulin regions, and *TNFAIP3* (50–52). Such genetic variant studies may indicate a possible explanation for the imprint of evolutionary history of these immune variations. Here, we performed a cohort study of CRC patients from Beijing, China and found that patients carrying the hIgG1-G396R variant possessed improved survival and attenuated clinical symptoms, highlighting hIgG1-G396R as an independently positive prognostic factor. This finding is of significance for clinical benefit in light of the low treatment response rate of CRC. CRC is highly heterogeneous, with only a small subset characterized as MSI (53). The status of MSI confers higher tumor mutational burden (TMB) and neoantigen load in tumors, which are crucial for the infiltration of effector immune cells and effective initiation of immune activation to potentiate antitumor activity in the TME (54). Recent immune checkpoint blockade treatment has proven to be an effective immunotherapeutic strategy for MSI CRC patients (55). However, 85% of CRC patients have MSS tumors with low to moderate TMB, and there were only limited applications of immune checkpoint inhibitors in MSS CRC patients. Thus, the core problem is how to establish antitumor immune machinery in the context of limited immunogenicity.

In this study, we found that hIgG1-G396R represses tumor progression, independently of MSI/MSS status. Moreover, it seems that the hIgG1-G396R in MSS CRC subpopulation contributes more clinical benefits in terms of longer PFS compared with its effects in MSI patients. This might be attributable to the impact of hIgG1-G396R on IgG1^+^ B cell activation and to a broader extent, the mobilization of various immune populations in the TME. Indeed, we found increased TAA-specific IgG^+^ B cells and plasma cells and elevated TAA-specific IgG antibodies in variant-harboring CRC patients and murine models. These are consistent with our early studies showing that hIgG1-G396R enhances IgG1^+^ B cell activation and differentiation to plasma cells by potentiating the phosphorylation of the IgG1 immunoglobulin tail tyrosine motif and lowering the IgG1-BCR activation threshold (17). Starting with the enhanced IgG^+^ B cell activity, more effector T cells and myeloid cells are further activated. Thus, the TME exhibits increased infiltration of CD8^+^ T cells and antigen-presenting cDC1s, supporting the establishment of potent antitumor immunity. Collectively, these findings suggest a model wherein hIgG1-G396R fuels immune responses against tumors in at least 4 ways: (a) elevated tumor-specific IgG1 production, which promotes avidity of macrophages, DCs, and NK cells toward IgG1-bound tumors (56, 57); (b) improved ADCP by macrophages and DCs; (c) enhanced presentation of TAAs and neoantigens to potentiate CD8^+^ T cell activation; and (d) potentiated formation of functional TLSs, in which switched memory B cells are enriched in responders and synergize with killer T cells for effective tumor cell targeting.

We expect that class-switched memory B cells might also play additional roles. For example, activated tumor-infiltrating B cells might secrete cytokines such as IFN-γ and TNF-α to fine-tune the immune context in tumors (58). In addition, hIgG1-G396R might alter the affinity of antibodies targeting TAAs and neoantigens, and in turn block immune evasion of tumor cells. Future studies will be important to explore these phenomena and other potential mechanisms, and determine whether hIgG1-G396R has similar effects on survival and progression in other cancer types.

Currently, the response rate of immunotherapy by immune checkpoint blockade still needs to be improved and the combination of immune checkpoint inhibitors with other treatments such as radiotherapy, chemotherapy, or other immune modulators such as myeloid cell activation or immune-suppression agents are emerging highlights of the clinical activity of investigational therapies (59, 60). Our findings here shed light on the potential value of enhancing class-switched IgG1^+^ memory BCR-mediated neoantigen targeting and IgG1 subtype antibody–mediated TAA presentation to replenish immune cell mobilization in the TME. Most importantly, our finding of a single genetic variant in IgG1^+^ B cells in colorectal tumorigenesis lays the foundation for designing novel cancer therapies. The favorable antitumor outcomes after adoptive transfer of highly reactive class-switched memory B cells in mice highlight the possibility of combined conventional T cell–targeting immunotherapy with adoptive human memory B cells, especially focusing on the TAA-specific IgG1^+^ memory B cells. Thus, our work suggests that IgG1^+^ memory B cells might serve as tuners for orchestrating broad and robust immunosurveillance and provide the notion that harnessing human IgG1^+^ memory B cells in the TME could be a powerful strategy for cancer immunotherapy.

## Methods

### CRC patients and healthy controls.

Samples from a total of 1006 CRC patients with histological verification by Peking University People’s Hospital and 583 healthy controls (HCs) were reanalyzed for this study. After surgery, the CRC samples and tumor-adjacent tissues were immediately collected and separated as aliquots in cryogenic vials. All tissues were preserved at –80°C before further treatments and assays. Basic features (sex, age, et al.), pathologic features (tumor location, tumor size, gross morphology, histological type, tumor differentiation, lymphovascular invasion, pathological TNM stage, clinical stage, et al.), and serum tumor markers (CEA, AFP, CA199, CA125, et al.) were documented in detail at Peking University People’s Hospital. TNM staging was performed following the instructions of American Joint Committee on Cancer (AJCC, 7th edition). OS was recorded and calculated from the time of surgery to the last follow-up or date of death due to CRC. PFS was defined the time from the first time of disease progression or death from any cause.

### TaqMan probe–based rs117518546 genotyping.

The genomic DNA of CRC patients and HCs were extracted from white blood cells. Genotyping of rs117518546 was performed using the TaqMan Genotyping Master Mix (Thermo Fisher Scientific) and SNP genotyping probes for rs117518546 (Thermo Fisher Scientific) following the manufacturer’s instructions and our published protocols (17).

### Reagents.

AOM, OVA, PI, and bovine serum albumin (BSA) were purchased from Sigma-Aldrich. DSS was purchased from MP Biomedicals. L-012 solution was purchased from WAKO chemicals. Mouse FcγR (CD16/CD32) blocking antibody, fixation, and permeabilization solution and mouse IFN-γ Cytometric Bead Array (CBA) were purchased from BD Biosciences. FLT3L was purchased from PeproTech. M-CSF was purchased from BioLegend. Collagenase IV was purchased from VETEC. DNase I was purchased from ROCHE. L-Glutamine and penicillin/streptomycin were purchased from Gibco. TB Green Premix Ex Taq II was purchased from Takara. Protein A/G agarose prepacked column, Fast Flow was purchased from Beyotime. Taq MasterMix was purchased from Tsingke. Hematoxylin staining solution was purchased from ZSGB Biotech. Formalin solution (10%) was purchased from Leagene Biotech. Tissue Grinder G50 was purchased from Coyote Bioscience. MEGAclear kit, MEGAshortscript transcription kit, and CellTrace Violet (CTV) were purchased from Invitrogen. HiPure Total RNA Mini Kit was purchased from Magen. cDNA Synthesis Kit was purchased from Thermo Fisher Scientific. TH-Z93 adjuvant was a gift from Yonghui Zhang (Tsinghua University). Antibody information is available in Table 1.

### Cell culture and transfection.

MC38, LLC, B16F10, and HEK 293T cells were purchased from the national Biomedical Cell Resource (BMCR, China). Generally, cells were cultured in complete DMEM medium containing 10% heat-inactivated FBS, 2 mM L-glutamine, and 100 U/mL penicillin/streptomycin. Lentivirus was constructed on the PHAGE backbone. MC38 and B16F10 cells were infected by lentivirus to stably express both fluorescent protein mCherry and exogenous membrane-bound OVA (MC38-mOVA, B16-mOVA). LLC cells were infected by lentivirus to stably express both fluorescent protein mCherry and exogenous membrane-bound m2e (influenza virus M2 extracellular domain) in 3 tandem repeats (LLC-m2e). Lentivirus-infected tumor cells were sorted through a BD FACSAria III based on the expression levels of mCherry.

### Mice.

*Rag1*^–/–^ (B6.129S7-*Rag1^tm1Mom^*/J, 002216) and μMT (B6.129S2-*Igh-6^tm1Cgn^*/J, 002288) mice were obtained from Hai Qi (Tsinghua University). mIgG2c-tailless and mIgG2c-G400R genetically modified mice were generated by CRISPR/Cas9-based gene manipulation in the WT C57BL/6J background, as described in detail below. Mice were maintained under specific pathogen–free conditions in the animal facility of Tsinghua University. Mouse experiments were performed according to the governmental and institutional guidelines to guarantee animal welfare.

### Construction of mIgG2C-G400R–knockin mouse and mIgG2C-tailless mouse.

mIgG2C-G400R and mIgG2C-tailless mice were constructed on the C57BL/6J mice background. Firstly, 2 optimal sgRNA target sequences were screened out from the CRISPR design website (https://zlab.bio/guide-design-resources): 5′-GCTCAGACCCTCCAAACTGT-3′ and 5′-AGGATGGATGGGCTTCTGCA-3′. The homology-directed repair (HDR) target gene template for mIgG2c-G400R KI was a DNA fragment of the mouse *IGHG2C* sequence, containing two 800-bp homology arms flanking the mIgG2C-G400R mutation site and mutated protospacer adjacent motif (PAM) sites. Cas9 mRNA and sgRNAs were transcribed into mRNA by MEGAshortscript transcription kit (Invitrogen) in vitro, and the transcription products were further purified by MEGAclear kit (Invitrogen). The knockin mixture for mIgG2c-G400R mouse manipulation was prepared in a total of 10 μL with final concentrations of 3 ng/μL sgRNA1, 3 ng/μL sgRNA2, 10 ng/μL Cas9 mRNA, and 10 ng/μL HDR template. The knockout mixture for mIgG2c-tailless mouse manipulation was prepared in a total of 10 μL with final concentrations of 3 ng/μL sgRNA1, 3 ng/μL sgRNA2 , and 10 ng/μL Cas9 mRNA. The mixtures were delivered to mouse zygotes through microinjection. The generated mouse was verified through sequencing mouse PCR products, and mouse genotyping primers are described in detail below. The mIgG2c-G400R mouse and mIgG2c-tailless mouse were backcrossed to C57BL/6J mice for at least 3 generations.

### Mouse genotyping.

Genomic DNA was extracted from a section of the mouse tail. PCR amplification was performed using Taq MasterMix. PCR amplicons were sequenced. Genotyping primers for mIgG2c-tailless and mIgG2c-G400R mice were forward, 5′-TCCTCCATTCCCTGAGCC-3′ and reverse, 5′-TGGTTCTTCTGGTCCGGAG-3′.

### Mouse immunization and ELISA.

Six-week-old sex-matched mIgG2c-tailless, WT, and mIgG2c-G400R mice were injected with 100 μg OVA in combination with 100 μg TH-Z93 adjuvant through the foot pad (42). Serum samples were collected via tail vein on day 0 and weekly after immunization to quantify OVA-specific antibody titers. Sera samples were stored at –80°C before further processing. To detect antibody levels, 2 μg/mL biotin-conjugated goat anti–mouse IgG or 2 μg/mL goat anti–mouse IgA was coated on the ELISA plate overnight at 4°C to capture antibodies. ELISA plates were blocked with 5% skim milk in PBS at 37°C for 1 hour, and then incubated with serum and colon explant culture medium or control culture medium for 2 hours. Peroxidase-conjugated immunoglobulin subclass–specific (IgG-, IgG1-, IgG2b-, IgG2c-, IgG3-, and IgA-specific) antibodies were further used for final detection. To detect OVA-specific antibodies, 2 μg/mL OVA in PBS was coated on the ELISA plate.

### Tumor growth and treatment.

For the MC38 cell inoculation–derived tumor model, 3 × 10^5^ MC38 cells were resuspended in 100 μL sterile PBS and injected subcutaneously into the right flank of mice. For the MC38-mOVA cell inoculation–derived tumor model, 2 × 10^6^ MC38-mOVA cells were also resuspended in sterile PBS and injected subcutaneously into the right flank of mice on week 6 after recall immunization with OVA. For the B16-mOVA cell inoculation–derived tumor model, 2 × 10^6^ B16-mOVA cells in sterile PBS were injected intravenously into mice on week 6 after recall immunization with OVA.

For CD8^+^ T cell depletion experiments, 200 μg anti-CD8a antibody (Ultra-LEAF Purified anti-mouse CD8a, BioLegend) was intraperitoneally injected into WT and mIgG2c-G400R mice on day –2, 3, 10, and 17. The effects of CD8^+^ T cell depletion were monitored via flow cytometry. Then, 5 × 10^5^ MC38 cells were resuspended in 100 μL sterile PBS and injected subcutaneously into the right flank of mice.

Tumor growth was measured every 2 days, and tumor size was calculated by length (*a*) and width (*b*): tumor size = 0.52 × *ab*^2^. Mice were sacrificed when tumor size reached 2000 mm^3^.

### AOM/DSS-induced CAC model.

The AOM/DSS-induced CAC model has been described previously (28, 29). Briefly, 10-week-old male WT or mIgG2c-G400R mice were injected intraperitoneally with a single dose of the organ-tropic carcinogen AOM (10 mg/kg of body weight) on day 0. Then, mice were treated with 2.5% DSS (molecular weight 35–50 kDa) in drinking water for 7 days in each turn, followed by recovery for 10 days with normal drinking water. Mice were monitored for weight loss twice a week. The serum samples were isolated at indicated time points in the figure legends. Mice were euthanized on week 16 after induction and the colons were isolated to assess the tumor generation.

### In vivo imaging of intestinal cancer-associated inflammation.

After isoflurane-supported anesthesia, mice were intraperitoneally injected with 25 mg/kg L-012 solution. Bioluminescence images of each mouse were obtained under isoflurane anesthesia using an IVIS Spectrum system (PerkinElmer). For quantitative analyses, the included Living Image software was utilized to calculate the intensity of bioluminescent signals at standardized regions of interest (ROIs) for each mouse.

### In vitro culture of colon explants.

The levels of IgG subclasses and IgA in colon explants were measured by ELISA. The method used for colon explant in vitro culture was described previously (61). Briefly, fresh colon was collected from AOM/DSS-induced mice and cut into pieces approximately 1 cm long. Then, the colon tissue was washed vigorously in sterile PBS 3 times, and then incubated in complete DMEM medium for 24 hours. To exclude unnecessary variables, colon pieces were also weighed before incubation, and colon pieces of equivalent wet weight were included into the in vitro culture procedure.

### H&E staining and IHC.

After euthanasia, colon specimens were immediately isolated from CAC-induced mice and fixed in 10% formalin solution overnight, and colon specimens were subsequently embedded in paraffin. Standard hematoxylin and eosin (H&E) staining was used to evaluate the pathological severity of colon tissues. For IHC analyses, slides were incubated with indicated primary antibodies in PBS containing 5% BSA overnight at 4°C. Then, streptavidin-HRP was added, and finally the slides were stained with both the DAB staining kit and hematoxylin staining solution (ZSGB-Bio). The slides were scanned using an Axio Scan Z1 (Zeiss) and all the images were analyzed via Zen imaging software (Zeiss).

### TAA microarray.

The OmicsArray TAA microarray (catalog PA003, https://www.genecopoeia.com/product/omicsarray-antigen-microarrays/) that we applied is a TAA panel developed by the Microarray Core Facility of the University of Texas Southwestern Medical Center. This microarray was developed by GeneCopoeia; iGeneBio (http://www.igenebio.com/) is the international distributor of GeneCopoeia in China and performed the TAA microarray experiments using the OmicsArray TAA chip. The TAA microarray contains cell cycle–associated proteins (p53, c-Myc, CDK2, BRCA1, BRCA2, et al.), glycoproteins (CA-125, CA15-3, CA19-9, CEA, LAMP-2, et al.), angiogenesis-related proteins (ferritin, THPO, VEGF-165), chemokines and cytokines (CCL2, CCL3, CXCL10, et al.) and other tumor-associated proteins (ANXA1, ERP29, et al.).

TAA-specific IgM, IgG1, IgG2b, and IgG2c subclasses in the serum samples from CAC-induced mice and TAA-specific IgM, IgG1, and IgG3 subclasses in the plasma samples from CAC patients were measured. The serum samples and plasma samples were diluted 50-fold and the secondary antibodies were diluted 1000-fold.

### RNA extraction and RT-qPCR.

After euthanasia, fresh colon specimens were immediately isolated and ground with Tissue Grinder G50 (Coyote Bioscience) to obtain colon tissue homogenates. A HiPure Total RNA Mini Kit was utilized to extract total RNA following manufacturer’s protocols. cDNA was then synthesized through reverse transcription according to the manufacturer’s protocol and examined by quantitative PCR analyses, and each group was detected in triplicate. All the qPCR primers are listed in detail in Supplemental Table 6.

### Adoptive transfer experiments.

TDLNs and spleen were prepared as single-cell suspensions and stained with biotin-conjugated anti-TER119, biotin-conjugated anti-CD3ε, biotin-conjugated anti-CD43 (BD Biosciences), and streptavidin microbeads (Miltenyi Biotec). Murine B cells were negatively purified utilizing AutoMacs Pro (Miltenyi Biotec). Spleens from C57BL/6J mice were prepared as single-cell suspensions and splenic CD4^+^ T cells and CD8^+^ T cells were stained with FITC-conjugated anti-CD4 antibody and PE-conjugated anti-CD8 antibody (BioLegend), and then purified by BD FACSAria III. Purified B cells were then adoptively transferred into μMT recipient mice (1 × 10^7^ B cells per mouse). Purified B cells and T cells were mixed in a 1:1 ratio and then adoptively transferred into *Rag1*^–/–^ recipient mice. The spleens of OVA-immunized mice were isolated and prepared as single-cell suspensions on week 2 after recall immunization with OVA. Single-cell suspensions were stained with DAPI, OVA–Alexa Fluor 561, FITC–anti–mouse B220 (BioLegend), and Alexa Fluor 647–conjugated Fab fragment anti–mouse IgG2c (Fc-specific, Jackson ImmunoResearch), and OVA-specific IgG2c^+^ live B cells were sorted via flow cytometry and adoptively transferred into μMT recipient mice (5 × 10^5^ B cells per mouse).

Twenty-four hours after adoptive transfer, recipient mice were inoculated with either MC38 cells (5 × 10^5^ cells per mouse) or MC38-mOVA cells (1 × 10^6^ cells per mouse) by subcutaneous injection. Tumor growth was monitored and recorded every 2 days. Mice were sacrificed when tumor size reached 2000 mm^3^.

For IgG reinfusion experiments, IgG was purified from the serum samples of either MC38-inoculated mice or OVA-immunized mice utilizing protein A/G agarose prepacked column (Fast flow, Beyotime). The purified IgG was further dialyzed overnight in sterile PBS. For reinfusion experiments in vivo, 200 μg purified IgG in 200 μL sterile PBS was intravenously injected into μMT recipient mice on day 0, 5, 10, and 15 after inoculation with 5 × 10^5^ MC38 tumor cells (62).

### Isolation of LPL and tumor-infiltrating lymphocytes.

For LPL preparation, the colon was collected with fat, mesentery, and intestinal contents carefully removed in cold HBSS buffer. The intestine was cut into 1-cm-long pieces and incubated in epithelial strip buffer (HBSS, 5 mM EDTA, 1 mM DTT, 5% FBS, 15 mM HEPES) at 37°C in a 200-rpm shaking incubator for 30 minutes. The colon pieces were washed twice and further incubated in enzyme solution (2 mg/mL collagenase IV and 0.1 mg/mL DNase I) for 45 to 60 minutes at 37°C. The LPL was released into the supernatant and collected into several vials as single-cell suspensions on ice for later usage. Subcutaneous tumors were isolated from tumor-bearing micee and cut into 1-mm pieces. The tumor pieces were digested with enzyme solution at 37°C with shaking for 1 hour. Digested products were filtered through 70-μm cell strainers. Filtered cell suspensions were stained after washing twice with MACS buffer (PBS, 1% FBS, 5 mM EDTA; ref. 63).

### Flow cytometry.

Murine primary cells were separated from the spleen, bone marrow, LNs, lamina propria, and subcutaneous tumor tissues and maintained as single-cell suspensions in MACS buffer. Single-cell suspensions were preincubated with anti–mouse CD16/CD32 (BD Biosciences) on ice for 30 minutes to block FcγRs, and then cells were stained with specific fluorescent dye–conjugated cell surface marker antibodies. Cells were also stained with either propidium iodide (PI) or DAPI to exclude the dead cells. After washing, cells were resuspended in MACS buffer and analyzed using either a BD LSRII or BD Symphony A5. Cell sorting was performed with the BD FACSAria III. All data were further processed with FlowJo (v10) software (TreeStar). For IgG2c^+^ plasma cell staining, primary cells were first blocked and stained with fluorochrome-conjugated rat anti–mouse B220 and CD138. After washing with MACS buffer, cells were fixed and permeabilized. Subsequently, cells were stained with Alexa Fluor 647–conjugated Fab fragment anti–mouse IgG2c, Fc specific (Jackson ImmunoResearch) on ice for 30 minutes before further FACS processing. The gating strategy for flow cytometry analyses is shown in Supplemental Figure 9.

### Restimulation of tumor-primed B cells.

B cells purified from TDLNs were activated in RPMI-1640 medium with 10 μg/mL LPS for 2 days (41). Activated B cells were used for immune-function analysis. The B cells were stimulated with irradiated MC38 cells. After 24 hours, the levels of secretion of IgG subclasses in supernatants were detected by ELISA.

### ADCP of tumor cells by FLT3L-DCs and BMDMs.

Primary cells were isolated from murine bone marrow and were then resuspended at the proper density. For FLT3L-DC induction, primary cells were cultured in complete IMDM medium together with 100 ng/mL FLT3L cytokine for 9 to 10 days. For BMDM induction, primary cells were cultured in complete RPMI-1640 medium together with 20 ng/mL M-CSF cytokine for 6 days (34).

MC38-mOVA cells or LLC-m2e cells were prepared as necrotic cells (at least 3 freeze-thaw cycles) and cocultured with either BMDMs or FLT3L-DCs as effector cells at a 1:1 ratio in the presence of specific antibody or antiserum. After coculture, flow cytometry and confocal fluorescence imaging methods were applied to detect phagocytosed tumor cells and the efficiency of phagocytosis was calculated.

After coculture, tumor cell–pulsed FLT3L-DCs (following described mentioned above) were evaluated for their ability to prime OVA-specific OT-I CD8^+^ T cells. Purified OT-I CD8^+^ T cells were stained with CTV following the instructions in the user manual. DCs were sorted and cocultured with OT-I CD8^+^ T cells at a 1:10 ratio. After 48 or 72 hours, cells were collected and stained with PI and for CD8. The levels of proliferation were determined by CTV dilution using the BD LSRII. DCs were able to cross-prime OT-I T cells to a greater extent when mediated by antibody, which was clarified by increased percentages of proliferating OT-I CD8^+^ T cells. On day 3, the CBA cytokine kit was applied to evaluate IFN-γ secretion by CD8^+^ T cells in supernatant following the manufacturer’s instructions (44).

To examine the efficiency of phagocytosis, an Olympus FLUOVIEW FV1000 confocal laser scanning microscope equipped with 4 lasers (405 nm, 473 nm, 557 nm, and 635 nm) for fluorescence excitation, 2 photomultiplier tubes (PMTs) for fluorescence detection, and a 10× objective lens were utilized. Images were processed and analyzed by Image Pro Plus software (Media Cybernetics).

### Data and materials availability.

Further information and requests for resources and reagents should be firstly directed to the lead contact, WL (liulab@tsinghua.edu.cn).

### Statistics.

Actuarial analyses on OS and PFS were performed based on the detailed follow-up information. Kaplan-Meier analyses were carried out to build the survival curves, and log-rank tests were employed to assess the significance of the difference. R software v4.0.2 (https://mirrors.tuna.tsinghua.edu.cn/CRAN/) was used to analyze the probability of cumulative 5-year and 100-month survival rates. Student’s *t* test or Wilcoxon’s signed-rank test was used to explore quantitative variables as appropriate. Pearson’s χ^2^ test or Fisher’s exact test was used to explore categorical variables as appropriate. Univariable and multivariable COX regression analyses were performed to identify prognostic factors. Pearson’s χ^2^ test or binary logistic regression analysis was used for statistical analysis of allelic and genotypic differences between HCs and CRC patients. Statistical analyses were performed by using Prism 8.0.2 (GraphPad) and SPSS 23.0 (IBM).

### Study approval.

The reuse and reanalyses of this CRC cohort for this study were approved by the Ethics Committee of Peking University People’s Hospital (2020PHB046-02, 2020PHB047-01) and the Ethics Committee of Tsinghua University (20200073). All experimental studies were approved with assurance identification numbers 15-LWL2 and 19-LWL1 by the Institutional Animal Care and Use Committee (IACUC) of Tsinghua University.

## Author contributions

WL and ZS conceived the project. BY, WL, ZS, and XC designed experiments. BY, XYW, LD, CY, WS, YZ, and QZ performed experiments. Zhen Zhang, CY, L Zhu, SZ, QW, L Zhao, YL, XZ, ZW, and SXL prepared CRC patient samples and healthy control samples, and collected clinical information. L Zhang and SQ analyzed scRNA-seq data. BY and Zhen Zhang analyzed and interpreted most of the data. JH and F Wu provided the anti-m2e IgG2c antibody and interpreted the related data. BY, XC, WL, ZS, and Zhen Zhang wrote the manuscript. LL, F Wang, WZ, XHZ, YY, SW, ZL, HQ, Zemin Zhang, and DMK reviewed and revised the manuscript.

## Supplementary Material

Supplemental data

## Figures and Tables

**Table 1:**
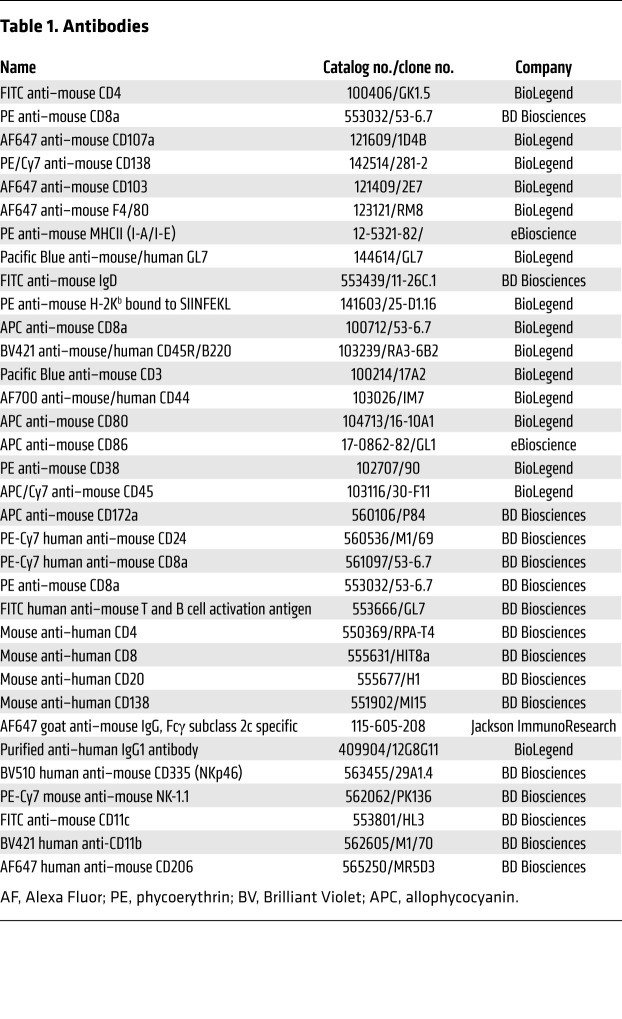
Antibodies

## Version 1 02/08/2022

In-Press Preview

## Version 2 03/15/2022

Electronic publication
