# Supplementary material for: An Asia-specific variant of human IgG1 represses colorectal tumorigenesis by shaping the tumor microenvironment
Source: J Clin Invest. 2022 Mar 15;132(6):e153454. doi: 10.1172/JCI153454 (PMC8920342; doi:10.1172/JCI153454)
Supplement: Supplemental data [file jci-132-153454-s129.pdf]

Supplemental figures

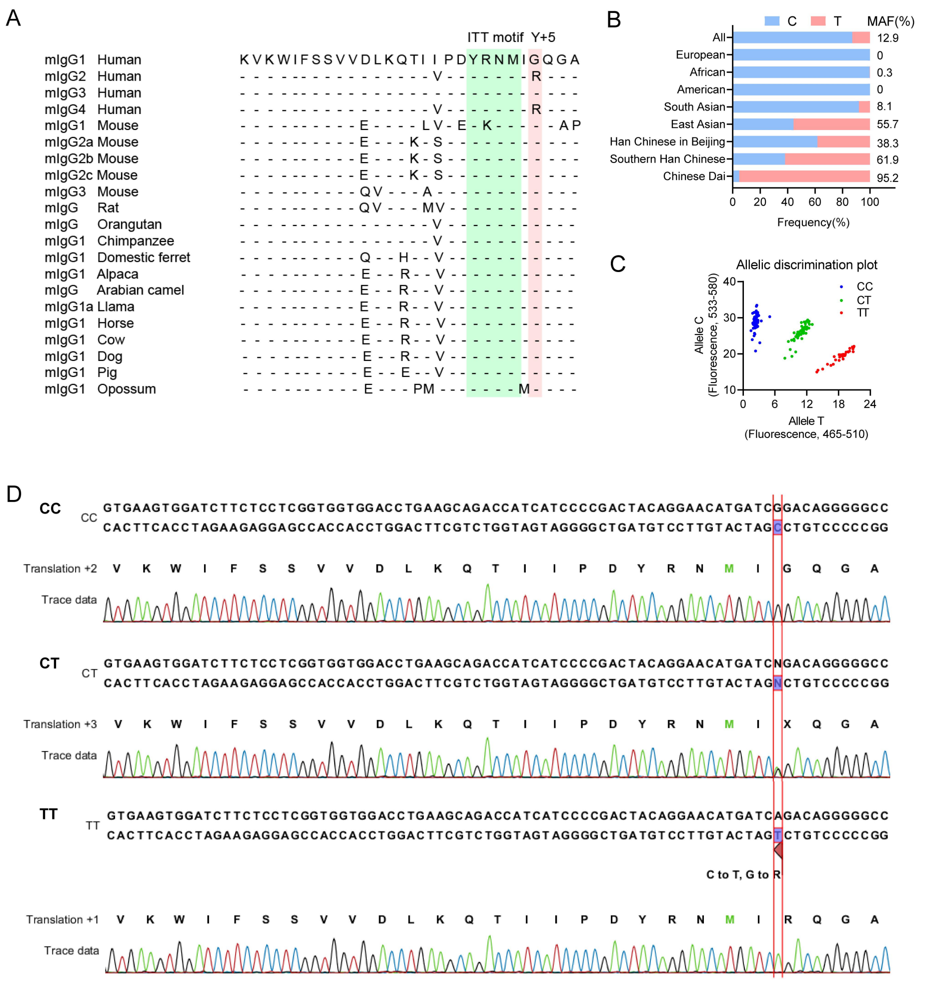

Supplemental Figure 1. The conserved cytoplasmic tail sequence of mIgG and the identification of the hIgG1-G396R variant.

(**A**) Amino acid sequences of the conserved tail of membrane-bound IgG in different species. (**B**) Global minor allele frequency (MAF) among populations in different regions are shown. (**C**) The distribution of three genotypes, including hIgG1-G396R homozygotes, heterozygotes and WT, detected by TaqMan-probe based genotyping. (**D**) The aligned target sequences of WT, hIgG1-G396R heterozygote and homozygote. The C/ T polymorphism are highlighted.

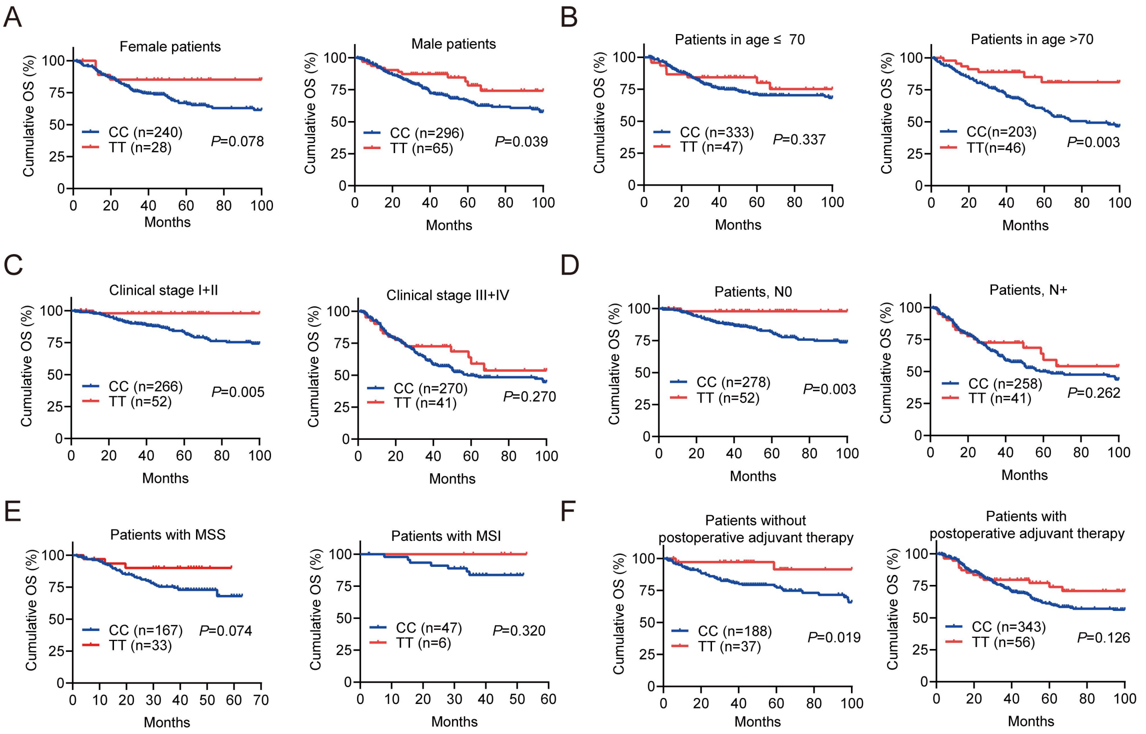

Supplemental Figure 2. hIgG1-G396R variant is consistently associated with prolonged OS.

(**A-F**) OS curves of 1006 CRC patients with different clinical treatments and clinical manifestations. Statistical significance was determined using a Log-rank test (A-F).

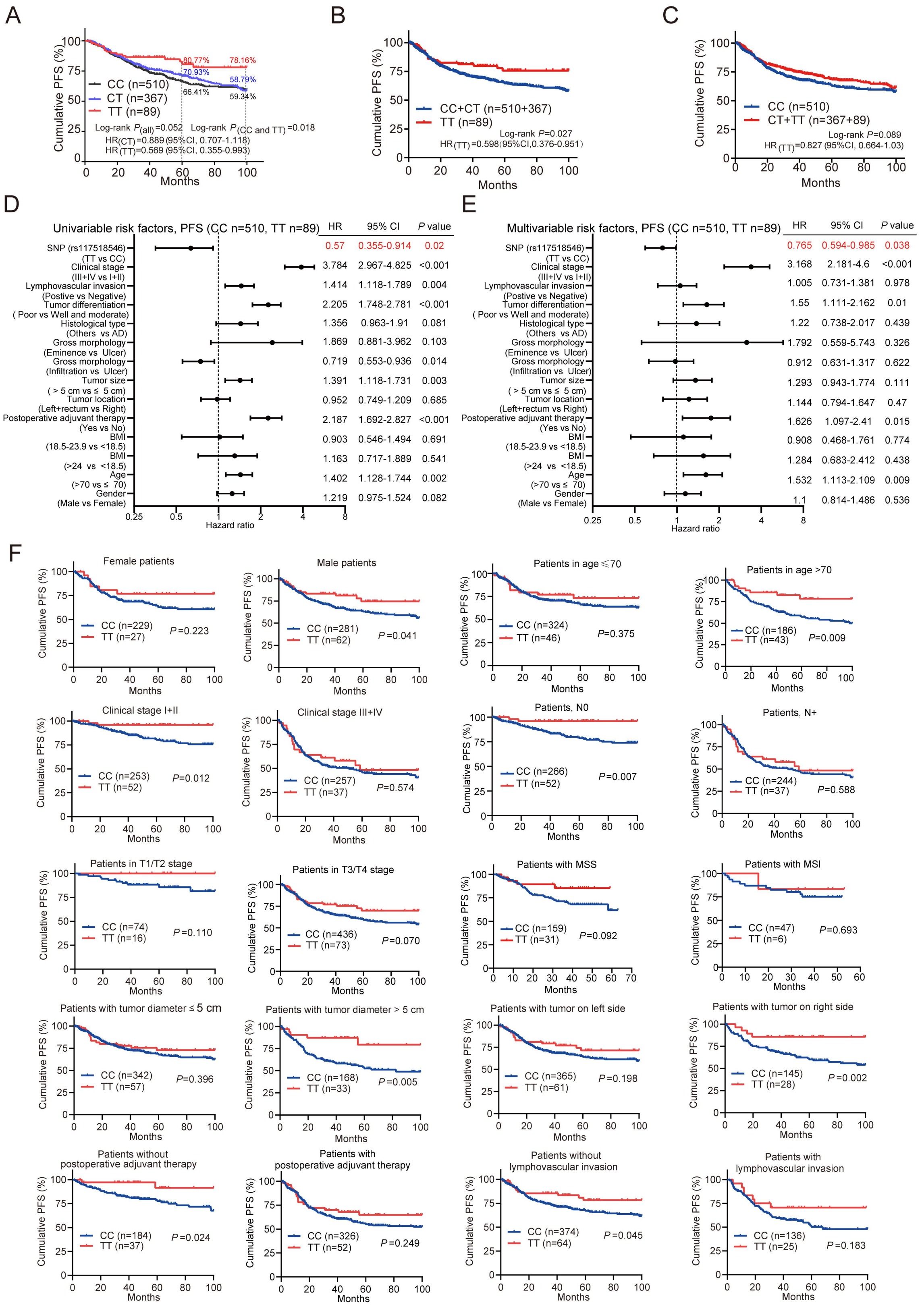

Supplemental Figure 3. hIgG1-G396R variant is associated with superior PFS and is an independent favorable prognosis marker for PFS.

(**A**) Survival curves of 966 CRC patients with available follow-up information of PFS were analyzed for the association of hIgG1-G396R with PFS. (**B-C**) Survival curves of 966 CRC patients grouped by recessive model (**B**) and dominant model (**C**). (**D**) Univariable and (**E**) multivariable COX regression analysis of PFS of 966 CRC patients and risk factors. (**F**) PFS curves of CRC patients with different treatments and clinical manifestations. Statistical significance was determined using a Log-rank test.

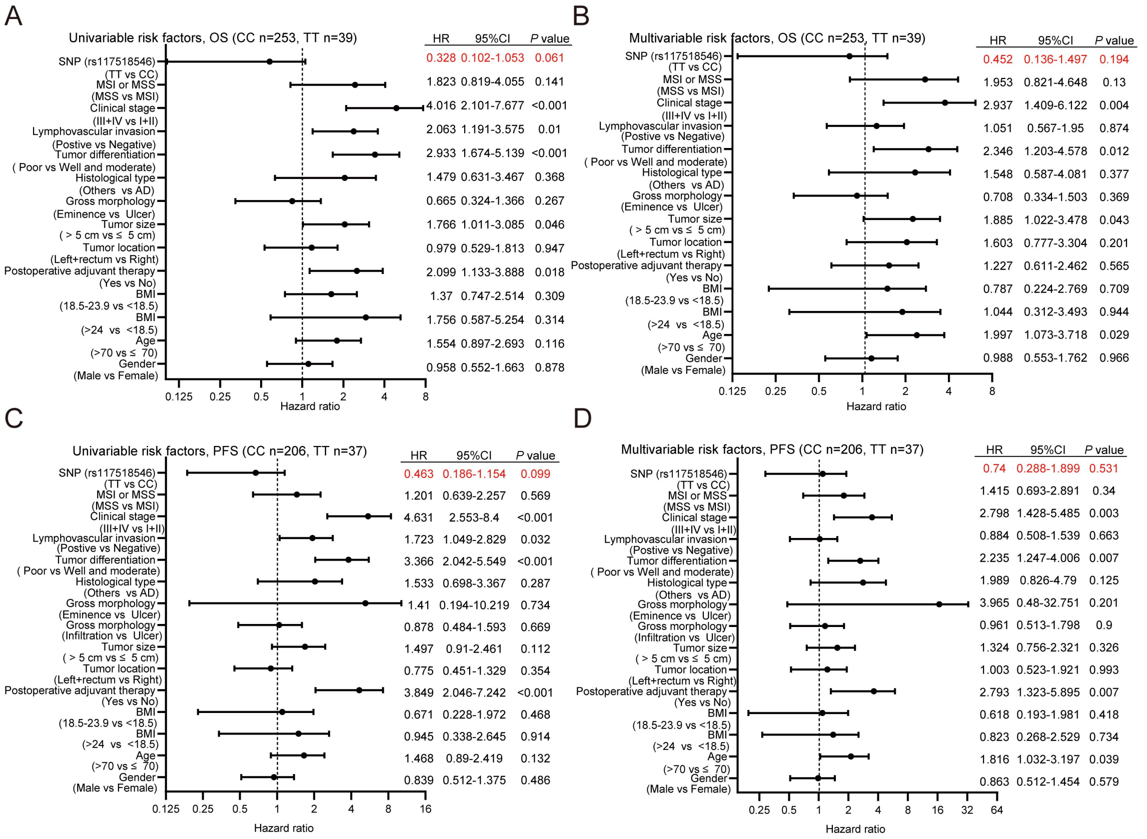

Supplemental Figure 4. Multivariable regression analyses of CRC patients with MSS/MSI information.

(**A-B**) Forest plot based on the results of (**A**) univariable risk analysis and (**B**) multivariable risk analysis of the risk factors associated with OS of CRC patients (n=292), which have MSI/MSS information records. (**C-D**) Forest plot based on the results of (**C**) univariable risk analysis and (**D**) multivariable risk analysis of the risk factors associated with PFS of CRC patients (n=243), which have available MSI/MSS information records and PFS records. HR, hazard ratio.

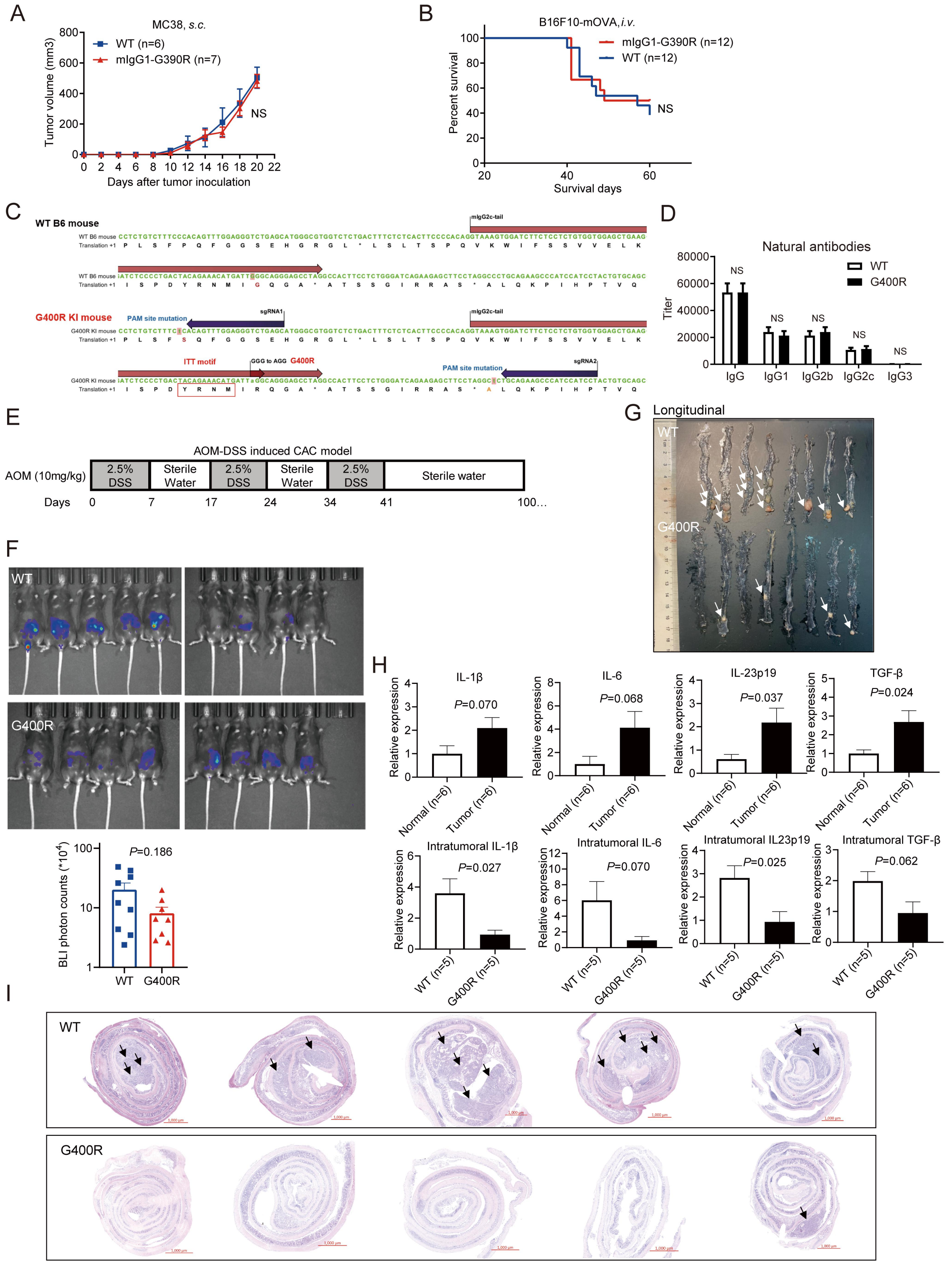

Supplemental Figure 5. The mIgG2c-G400R knock-in mice, manipulating by CRISPR-Cas9 system, exhibit significantly ameliorated colon tumorigenesis.

(**A**) Tumor growth of MC38 cells in WT (n=6) and mIgG1-G390R (n=7) mice. (**B**) Survival curves of OVA-immunized WT (n=12) and mIgG1-G390R (n=12) mice after

intravenous injection of B16F10-mOVA tumor cells. (**C**) Alignment of the *IGHG2C* DNA sequence from NCBI database with the sequencing data of the mIgG2c-G400R knock-in mouse. Annotated are the sgRNA target sequences, mIgG2c-tail and mIgG2c-G400R variant site. (**D**) Basal levels of natural IgG, IgG1, IgG2b, IgG2c and IgG3 antibodies in the serum samples of untreated 6-week-old WT (n=6) and mIgG2c-G400R (n=6) mice. Statistical significance was determined using an unpaired two-tailed t-test (B). Mean± SEM. NS, not significant. (**E**) A schematic overview of the AOM-DSS induced CAC model. (**F**) Bioluminescent images obtained at week 6 after AOM-DSS treatment following intraperitoneal injection of L-012 solution. (**G**) Representative longitudinal images of tumor burden in the colon specimens from CAC induced mice. Tumors indicated by white arrows. (**H**) RT-qPCR analyses of intratumoral cytokine mRNAs from colon tumors and matched normal colons of CAC-induced WT mice and mIgG2c-G400R mice. (**I**) Representative images of H&E-stained colon cross-sections from CAC induced WT mice (n=5) and mIgG2c-G400R mice (n=5). Scale bar, 1000 μm. One of three representative experiments is shown. Statistical significance was determined using two-way ANOVA (A, B) or unpaired two-tailed t-test (D, F, H). Mean± SEM. NS, not significant.

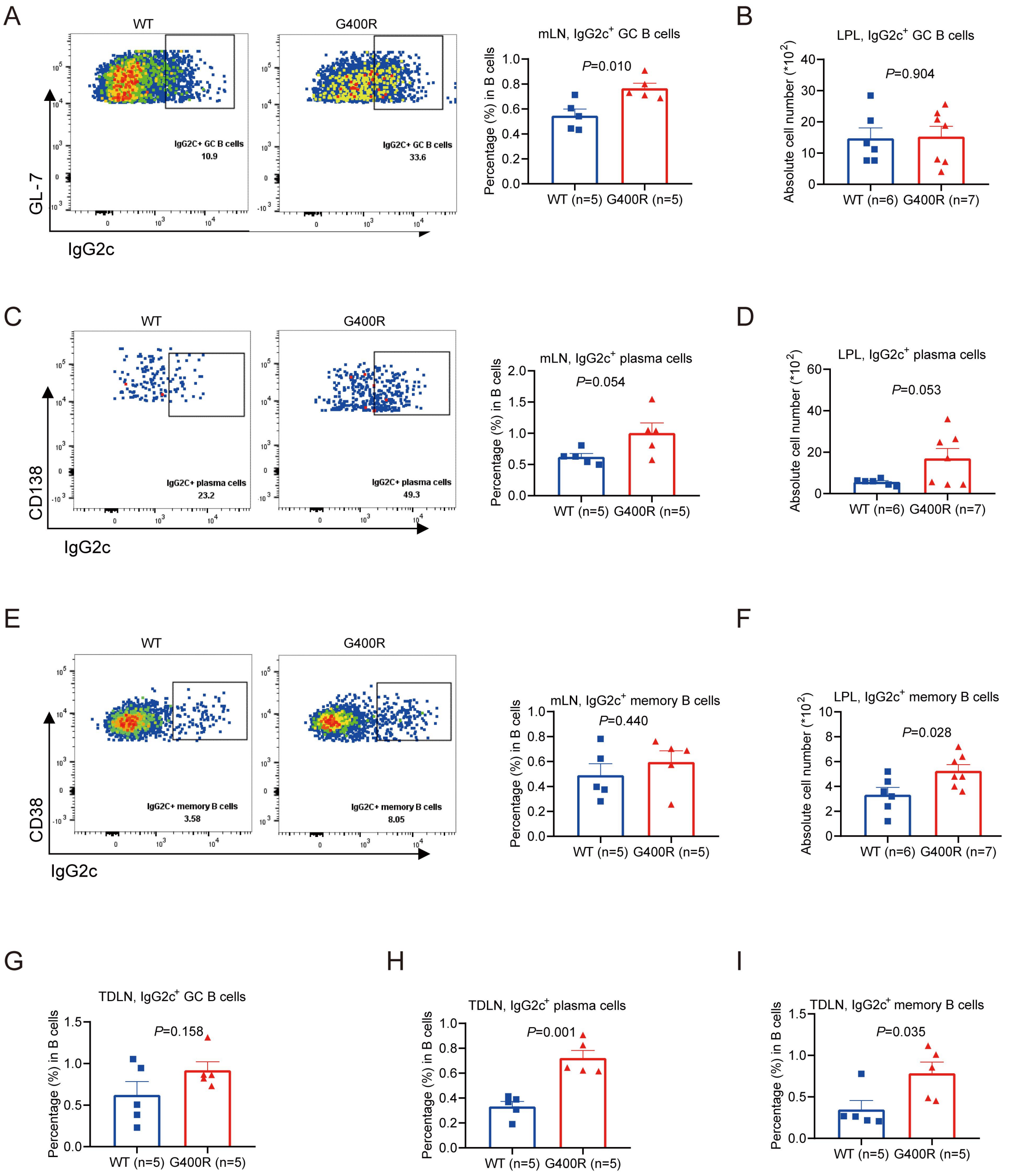

Supplemental Figure 6. mIgG2c-G400R promotes B cell differentiation upon tumor cell inoculation.

(**A--F**) The relative percentages of IgG2c$^+$ GC B cells in (**A**) mLNs and (**B**) lamina propria (LP), plasma cells in (**C**) mLNs and (**D**) LP, memory B cells in (**E**) mLNs and (**F**) LP from CAC-inducing mIgG2c-G400R mice in comparison to WT mice. (**G-I**) The relative percentages of IgG2c$^+$ GC B cells (**G**), plasma cells (**H**) and memory B cells (**I**) in TDLNs from MC38 cell-inoculating mice. One of three representative experiments is shown. Statistical significance was determined using an unpaired two-tailed t-test.

Mean± SEM.

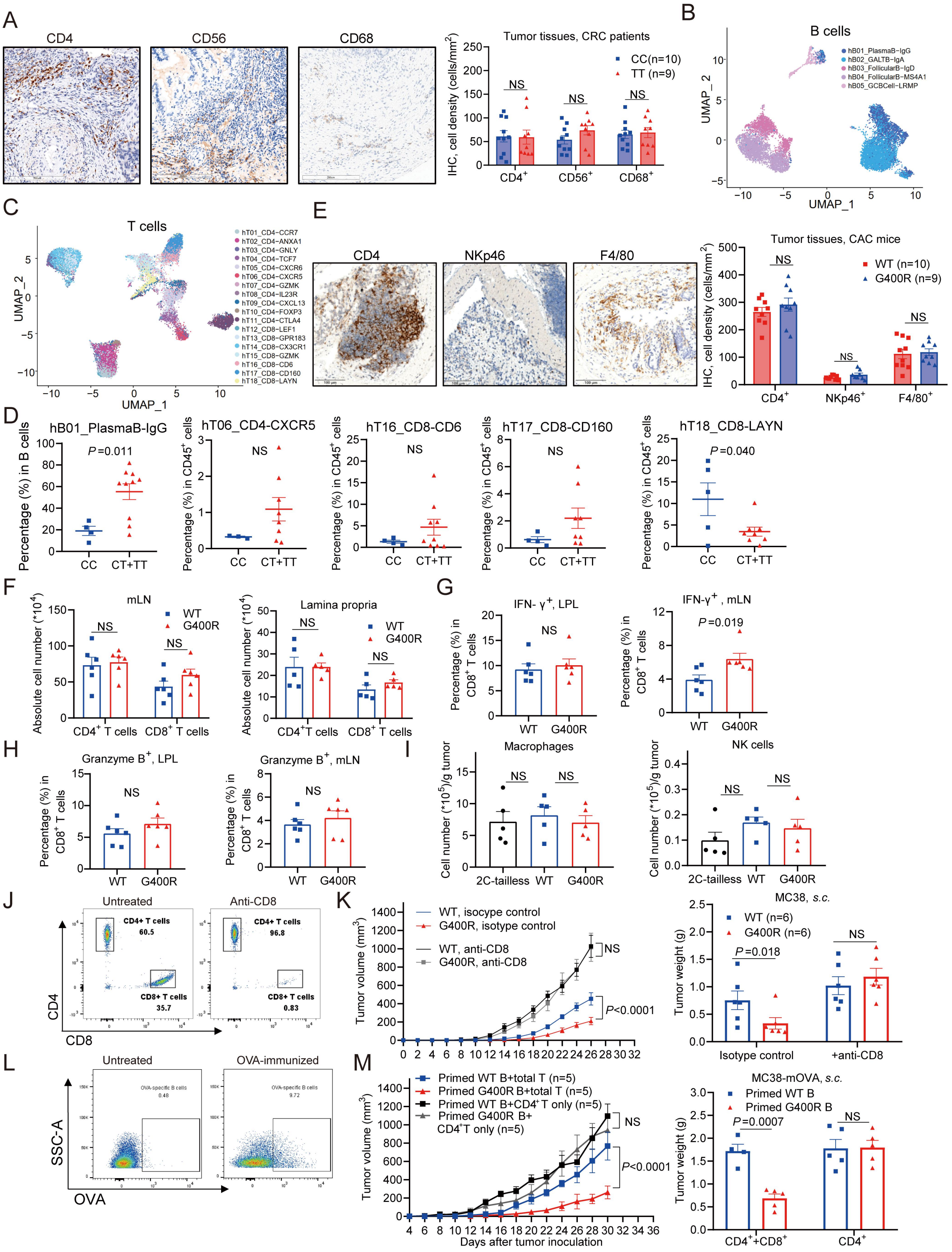

Supplemental Figure 7. Altered immune microenvironment in mIgG2c-G400R mice.

(**A**) Representative images of colon specimens from CRC patients immunohistochemically stained with CD4, CD56 and CD68. Scale bar, 200 μm. The

numbers of $CD4^+$ T cells, NK cells, macrophages per mm^2 are shown. (**B-C**) Representative UMAP plot showing the clusters of (**B**) B cells and (**C**) T cells from 18 CRC patients analyzed by scRNA-seq. (**D**) The proportions of tumor-infiltrating *IgG*$^+$ plasma cells within total B cells, *CXCR5*$^+$ T follicular helper cells, *CD6*$^+$ tumor-resident memory T cells, *CD160*$^+$ intraepithelial $CD8^+$ T cells and *LAYN*$^+$ exhausted T cells within total $CD45^+$ cells from the scRNA-seq results. (**E**) Representative IHC staining images of the colon tumor specimens from CAC-induced mice. Scale bar, 100 μm. The numbers of $CD4^+$ T cells, NK cells, macrophages per mm^2 are shown. **(F)** Quantification of $CD4^+$ T cells and $CD8^+$ T cells in the mLN and lamina propria of CAC mice, basing on the results of flow cytometry. (**G-H**) Flow cytometric analyses of $CD4^+$ T cells, $CD8^+$ T cells, IFN-γ secreting and GZMB secreting $CD8^+$ T cells. (**I**) Quantification of tumor-infiltrating macrophages and NK cells of MC38-mOVA cell inoculated mice, basing on the results of flow cytometry. (**J**) The number of $CD8^+$ T cells in the blood after anti-CD8a antibody dominated $CD8^+$ T cell depletion was detected via flow cytometry and representative flow plots are shown. (**K**) WT mice and mIgG2c-G400R mice in the isotype control and anti-CD8 groups were subcutaneously injected with MC38 cells. Tumor growth curves and tumor weight are shown. (**L**) *Rag1*$^{-/-}$ mice were transferred with either $CD4^+$ and $CD8^+$ T cells or $CD4^+$ T cells alone with OVA-specific B cells, followed by MC38-mOVA cell inoculation. (**M**) Tumor growth curves and tumor weight are shown. Statistical significance was determined using an unpaired two-tailed t-test and two-way ANOVA (K, M). Mean± SEM. NS, not significant.

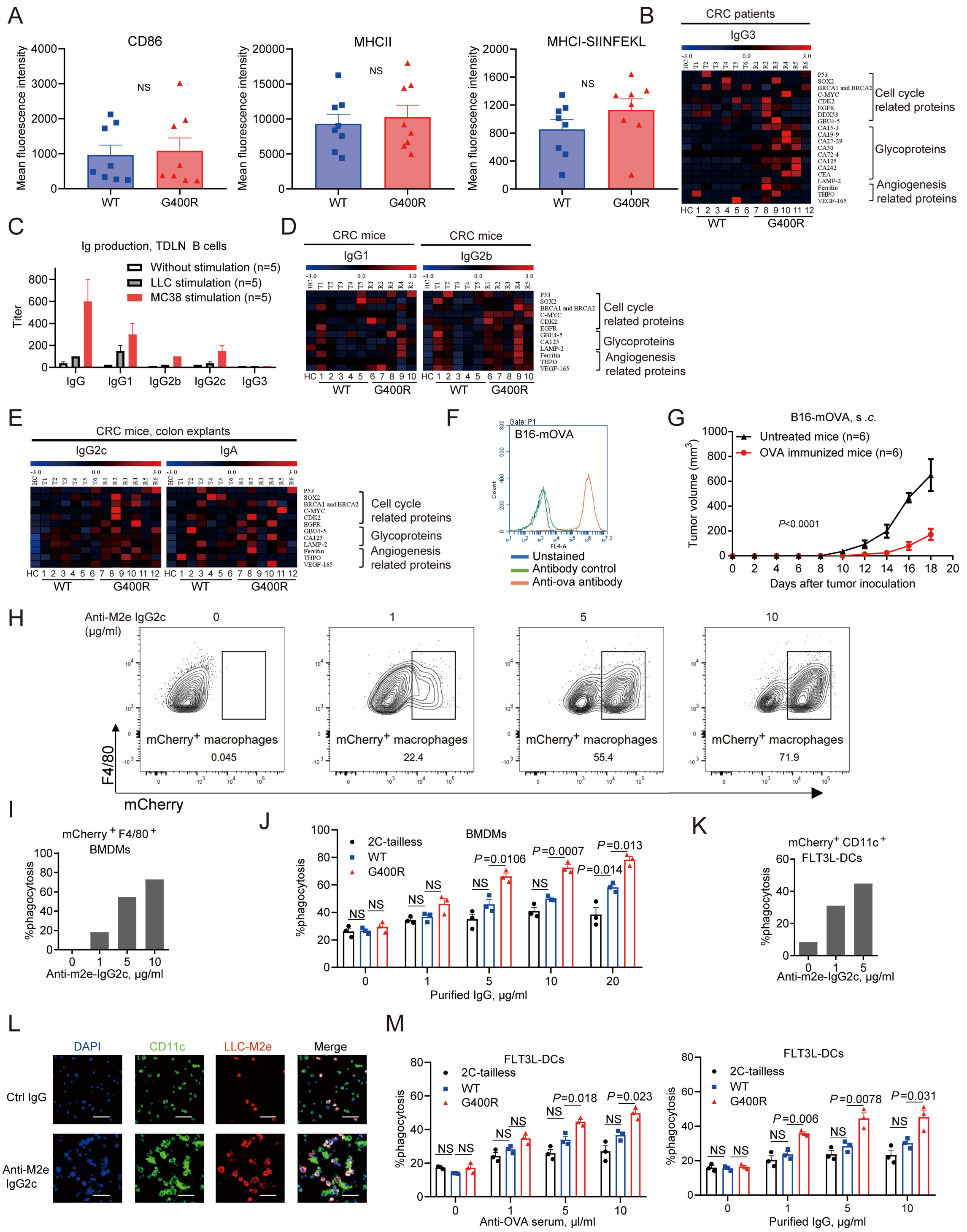

Supplemental Figure 8. Enhanced tumor-specific antibody production and ADCP efficiency mediated by phagocytes, occurring with both hIgG1-G396R and mIgG2c-G400R variant.

(**A**) Expression of CD86, MHC II, MHC I-SIINFEKL by tumor-infiltrating IgG2c^{+} B cells at day 3 post MC38-mOVA tumor cell inoculation, detected by flow cytometry. (**B**)

Heatmap showing the levels of IgG3 in the plasma of healthy donor (n=1), WT CRC patients (n=6) and hIgG1-G396R homozygous CRC patients (n=6). (**C**) Secretion of IgG subclasses by activated TDLN B cells in stimulation with cell medium, irradiated LLC cells or MC38 cells. (**D**) Heatmap showing the levels of IgG1 and IgG2b in the sera from untreated mouse (n=1), CAC-induced WT mice (n=5) and mIgG2c-G400R mice (n=5). (**E**) Heatmap showing the levels of IgA and IgG2c in the colon explants from WT CAC mice (n=6), mIgG2c-G400R CAC mice (n=6) and cell medium. (**F**) OVA expression in the surface of B16-mOVA cells detected by flow cytometry. (**G**) The tumor growth curves of B16-mOVA cells in untreated mice and OVA-immunized mice. (**H**) Representative flow cytometry plots to compare the ADCP activities of BMDMs with the presence of anti-M2e IgG2c antibody. (**I**) Fractions of F4/80$^+$ mCherry$^+$ cells after co-culture with anti-M2e IgG2c antibody-coated LLC-M2e cells, determined by flow cytometry. (**J**) Comparison of BMDMs mediated ADCP activities using purified IgG, prepared from the anti-OVA serum of OVA-immunized mice. (**K**) Comparison of FLT3L-DCs mediated ADCP activities using anti-M2e IgG2c and control IgG, detected by flow cytometry. (**L**) Representative confocal fluorescence images showing the process of phagocytosis mediated by FLT3L-DCs. Nuclei marked by DAPI (blue), DCs were stained with CD11c-FITC (green) and tumor cells labeled by mCherry (red). Scale bar, 50 μm. (**M**) Comparison of FLT3L-DCs mediated ADCP activities using anti-OVA serum and purified IgG, detected by flow cytometry. One of three representative experiments is shown. Statistical significance was determined using two-way ANOVA (G) and one-way ANOVA. (J, M). Mean± SEM. NS, not significant.

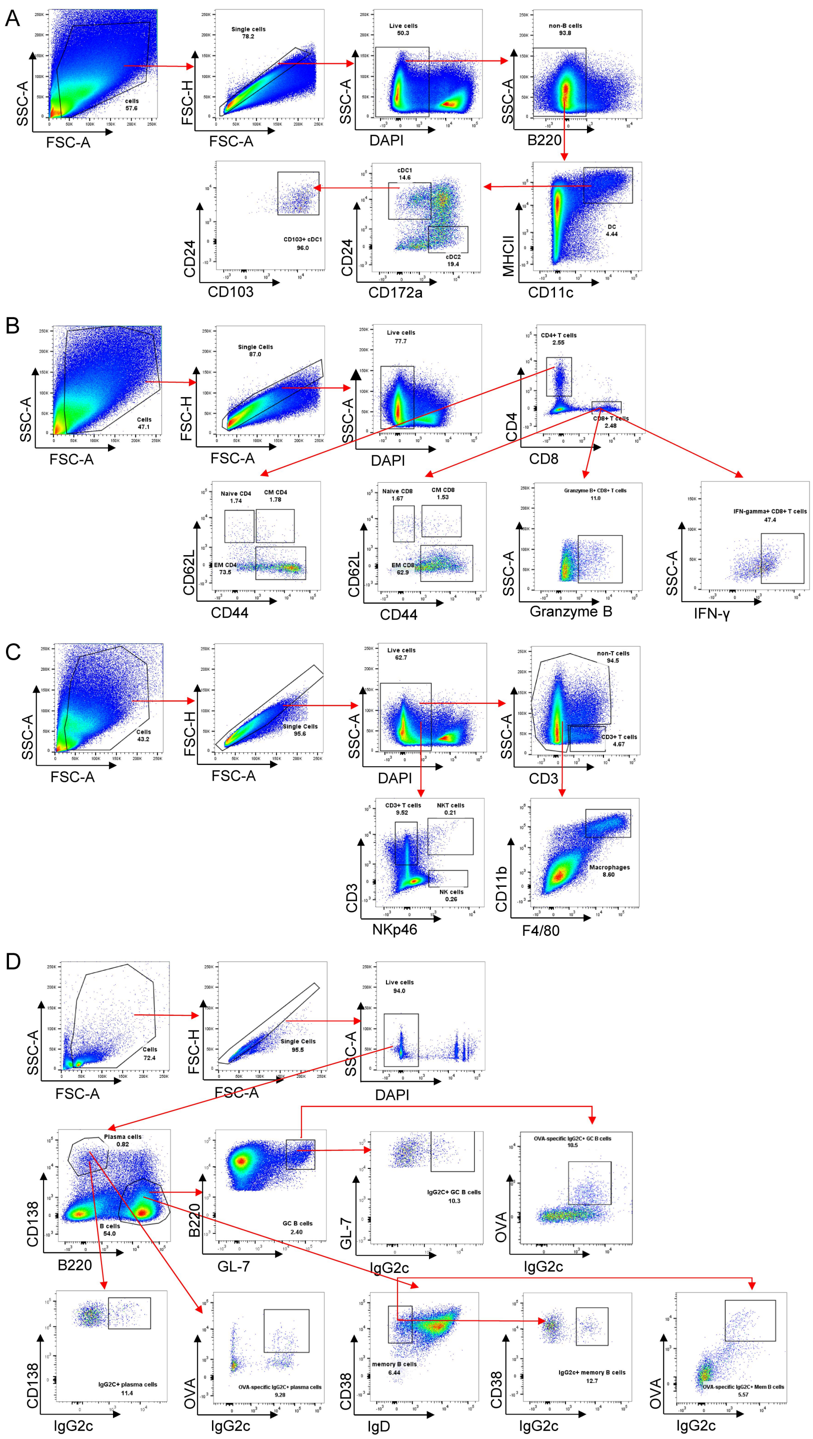

Supplemental Figure 9. Flow cytometry gating strategy for DCs (A), T cells (B), NK cells, macrophages (C) and B cell subsets (D)

Supplementary tables.

Characteristics	Number	%
**Gender**		
Female	427	42.4
Male	579	57.6
**Age at diagnosis (years)**		
Median	67	
Range	58-76	
**BMI**		
Median	23.5	
Range	21.2-26	
**Tumor size (cm)**		
Median	4	
Range	4-6	
**Tumor location**		
Ascending colon	241	24
Transverse colon	50	5
Descending colon	46	4.6
Sigmoid colon	247	24.6
Rectum	422	41.9
**Gross morphology**		
Ulcer type	720	71.6
Eminence type	272	27
Infiltration type	14	1.4
**Histological type**		
Adenocarcinoma	910	90.5
Other*	96	9.5
**Tumor differentiation**		
Well and Moderate	783	77.8
Poor	223	22.2
**Lymphovascular invasion**		
Positive	728	72.4
Negative	276	27.4
**T (tumor stage)**		
T1	24	2.4
T2	121	12
T3	368	36.6
T4	493	49
**N (regional lymph node)**		
N0	538	53.5
N1	261	25.9
N2	207	20.6
**M (metastasis)**		
M0	909	90.4

Characteristics	Number	%
M+	97	9.6
**Clinical stage**		
I	120	11.9
II	401	39.9
III	384	38.2
IV	101	10
**Postoperative adjuvant therapy**		
Yes	373	37.1
No	620	61.6
Unknown	13	1.3
**CEA (ng/ml)**		
Median	4.2	
Range (min to max)	0.12-1000	
**AFP (ng/ml)**		
Median	2.4	
Range (min to max)	0.18-24.57	
**CA199 (U/ml)**		
Median	12.5	
Range (min to max)	0.5-1000	
**CA125 (U/ml)**		
Median	9.6	
Range (min to max)	0-882.6	

* Mucinous adenocarcinoma, neuroendocrine carcinoma, signet ring cell carcinoma

Supplemental Table 1. Baseline clinical characteristics of CRC Patients (n=1006).

TNM staging was performed according to AJCC 's (8th edition) instructions.

SNP rs117518546			HCs (%)	CRC patients (%)	OR	95% CI	*P*
**Allelic**		**C**	813 (69.73)	1450 (72.07)	0.893	0.762-1.046	0.086
		**T**	353 (30.27)	562 (27.93)			
**Genotypic**	**Dominant**	**CC**	300 (51.46)	537 (53.38)	0.926	0.755-1.136	0.246
		**CT+TT**	283 (48.54)	469 (46.62)			
	**Recessive**	**CC+CT**	513 (87.99)	913 (90.76)	0.747	0.538-1.037	0.049*
		**TT**	70 (12.01)	93 (9.24)			
	**Additive**	**CC**	300 (51.46)	537 (53.38)	0.902	0.775-1.049	0.180
		**CT**	213 (36.53)	376 (37.38)			
		**TT**	70 (12.01)	93 (9.24)			

	HCs	CRC Patients
**Sample size**	583	1006
**Male**	335 (57.46%)	579 (57.55%)
**Female**	248 (42.54%)	427 (42.45%)
**Age (Mean± SEM)**	35.18±18.57	66.26±12.41

*P value < 0.05

OR, odds ratio; CI, confidence interval

Supplemental Table 2. The hIgG1-G396R variant allele frequency in HCs and CRC patients

rs117518546, OS	Univariable COX regression analysis			Multivariable COX regression analysis		
**Model**	**HR**	**95% CI**	***P* value**	**HR**	**95% CI**	**P value**
**Additive (CT vs CC)**	0.914	0.723-1.156	0.454	0.86	0.669-1.107	0.242
**Additive (TT vs CC)**	0.505	0.303-0.844	0.009*	0.467	0.27-0.81	0.007*
**Recessive (TT vs CC+CT)**	0.525	0.317-0.868	0.012*	0.497	0.289-0.853	0.011*
**Dominant (CT+TT vs CC)**	0.834	0.665-1.045	0.115	0.78	0.612-0.994	0.045*

rs117518546, PFS	Univariable COX regression analysis			Multivariable COX regression analysis		
**Model**	**HR**	**95% CI**	***P* value**	**HR**	**95% CI**	**P value**
**Additive (CT vs CC)**	0.889	0.707-1.118	0.315	0.874	0.684-1.116	0.28
**Additive (TT vs CC)**	0.569	0.355-0.913	0.02*	0.591	0.358-0.977	0.04*
**Recessive (TT vs CC+CT)**	0.598	0.376-0.951	0.03*	0.625	0.382-1.023	0.062
**Dominant (CT+TT vs CC)**	0.827	0.664-1.03	0.09	0.821	0.649-1.037	0.098

*P value < 0.05

Supplemental Table 3. Univariable and multivariable COX regression analyses to investigate the effects of hIgG1-G396R variants on OS or PFS.

	All cases	CC	CT	TT	*P*	CC	TT	*P*
**Gender, n(%)**								
Female	427	240(56.2)	159(37.2)	28(6.6)	0.032*	240(89.6)	28(10.4)	0.009*
Male	579	297(51.3)	217(37.5)	65(11.2)		297(82)	65(18)	
**Age, n(%)**								
≤70	587	333 (56.7)	207(35.3)	47(8.0)	0.031*	333(87.6)	47(12.4)	0.037*
>70	419	204(48.7)	169(40.3)	46(11.0)		204(81.6)	46(18.4)	
**BMI, n(%)**								
<18.5	61	27(44.3)	27(44.3)	7(11.5)	0.47	27(79.4)	7(20.6)	0.568
18.5-23.9	542	288(53.1)	207(38.2)	47(8.7)		288(86)	47(14)	
>24	355	196(55.2)	123(34.6)	36(10.1)		196(84.5)	36(15.5)	
**Native place distribution, n(%)**								
Northern Region	823	475(57.7)	302(36.7)	46(5.6)	<0.001*	475(91.2)	46(8.8)	<0.001*
Southern Region	183	62(33.9)	74(40.4)	47(25.7)		62(56.9)	47(43.1)	
**Tumor location, n(%)**								
Right colon	291	156(53.6)	105(36.1)	30(10.3)	0.71	156(83.9)	30(16.1)	0.531
Left colon and rectum	715	381(53.3)	271(37.9)	63(8.8)		381(85.8)	63(14.2)	
**Tumor size, n(%)**								
≤5cm	526	278(52.9)	199(37.8)	49(9.3)	0.94	278(85)	49(15)	0.87
>5cm	480	259(54)	177(36.9)	44(9.2)		259(85.5)	44(14.5)	
**Gross morphology, n(%)**								
Ulcer type	720	386(53.6)	269(37.4)	65(9)	0.73	386(85.6)	65(14.4)	0.568
Eminence type	272	142(52.2)	102(37.5)	28(10.3)		142(83.5)	28(16.5)	
Infiltration type	14	9(64.3)	5(35.7)	0(0)		9(100)	0(0)	
**Histological type, n(%)**								
Adenocarcinoma	910	487(53.5)	338(37.1)	85(9.3)	0.88	487(85.1)	85(14.9)	0.827
Other**	96	50(52.1)	38(36.9)	8(8.3)		50(86.2)	8(13.8)	
**Tumor differentiation, n(%)**								

	All cases	CC	CT	TT	*P*	CC	TT	*P*
Well and Moderate	783	414(52.9)	289(36.9)	80(10.2)	0.14	414(83.8)	80(16.2)	0.053
Poor	223	123(55.2)	87(39.0)	13(5.8)		123(90.4)	13(9.6)	
**Lymphovascular invasion, n(%)**								
Positive	728	390(53.6)	270(37.1)	68(9.3)	0.96	390(85.2)	68(14.8)	0.943
Negative	276	146(52.9)	105(38)	25(9.1)		146(85.4)	25(14.6)	
**T, n(%)**								
T1+T2	145	76(52.4)	53(36.6)	16(11)	0.72	76(82.6)	16(17.4)	0.442
T3+T4	861	461(53.5)	323(37.5)	77(8.9)		461(85.7)	77(14.3)	
**N, n(%)**								
N0	538	278(51.7)	208(38.7)	52(9.7)	0.51	278(84.2)	52(15.8)	0.46
N+	468	259(55.3)	168(35.9)	41(8.8)		259(86.3)	41(13.7)	
**M, n(%)**								
M0	909	478(52.6)	343(37.7)	88(9.7)	0.18	478(84.5)	88(15.5)	0.098
M+	97	59(60.8)	33(34)	5(5.2)		59(92.2)	5(7.8)	
**Clinical stage, n(%)**								
I+II	521	266(51.1)	203(39)	52(10)	0.29	266(83.6)	52(16.4)	0.256
III+IV	485	271(55.9)	173(35.7)	41(8.5)		271(86.9)	41(13.1)	
**CEA(ng/ml), median(range)**	4.2(0.12-1000)	4.4(0.2-1000)	3.8(0.2-1000)	4.4(0.12-196.1)	0.44	4.4(0.2-1000)	4.4(0.12-196.1)	0.2
**AFP(ng/ml), median(range)**	2.4(0.18-24.57)	2.4(0.18-24.57)	2.3(0.24-23.12)	2.6(0.21-10.67)	0.62	2.4(0.18-24.57)	2.6(0.21-10.67)	0.685
**CA199(U/ml), median(range)**	12.5(0.5-1000)	12.5(0.6-1000)	12.3(0.5-1000)	13.4(0.6-1000)	0.61	12.5(0.6-1000)	13.4(0.6-1000)	0.381
**CA125(U/ml), median(range)**	9.6(0-882.6)	9.9(0-326.1)	9.3(0-882.6)	9.3(0-209)	0.68	9.9(0-326.1)	9.3(0-209)	0.514

* P value < 0.05; ** Mucinous adenocarcinoma, neuroendocrine carcinoma, signet ring cell carcinoma

Supplemental Table 4. Characteristics of CRC patients in different genotypes (n=1006).

CRC patients, number	rs117518546 genotype
P0413	CC
P1228	CC
P0411	CC
P0825	CC
P0202	CC
P0720	CC
P0123	CT
P1212	CT
P0309	CT
P0104	CT
P0305	CT
P0323	CT
P0408	CT
P0410	CT
P0613	CT
P0728	CT
P1026	CT
P1025	TT

Supplemental Table 5. Genotypes of 18 CRC patients involved in scRNA-seq analyses.

The rs117518546 genotypes of 18 CRC patients using in the scRNA-seq assays are shown, which were verified via either Taqman-probe-based genotyping or bulk WES.

Species	Target gene	Sequences (5'-3')	
Human	*GAPDH*	Forward	ACCTCAACTACATGGCTGAGAAC
		Reverse	CATGGTGGTGAAGACGCCAG
	IGHG1	Forward	AATGGGCAGCCGGAGAACAACT
		Reverse	TGCTCTTGTCCACGGTGAGCTT
	IGHG2	Forward	AATGGGCAGCCGGAGAACAACT
		Reverse	TGCTCTTGTCCACGGTGAGCTT
	IGHG3	Forward	TAAGCCCACCCCAAAGGCCAAA
		Reverse	ACCGTGGGCATGTGTGAGTTGT
	IGHG4	Forward	AATGGGCAGCCGGAGAACAACT
		Reverse	TTGTCCACGGTTAGCCTGCTGT
	IGHA	Forward	CGCTGGCCTTCACACAGAA
		Reverse	CGCCATGACAACAGACACA
	CXCL12	Forward	ACTGGGTTTGTGATTGCCTCTGAA
		Reverse	GGAACCTGAACCCCTGCTGTG
	CXCL13	Forward	GAGGCAGATGGAACTTGAGC
		Reverse	CTGGGGATCTTCGAATGCTA
	CCL19	Forward	CCAGCCCCAACTCTGAGTG
		Reverse	ATCCTTGATGAGAAGGTAGTGGA
	CCL21	Forward	CGCAGCTACCGGAAGCAG
		Reverse	CTGCCTGAGAGCGCTTGC
Mouse	*GAPDH*	Forward	AGTATGACTCCACTCACGGCAA
		Reverse	TCTCGCTCCTGGAAGATGGT
	TGFB	Forward	AGAGAAGAACTGCTGTGTGC
		Reverse	GGGTTGTGTTGGTTGTAGAG
	IL1B	Forward	CAACCAACAAGTGATATTCTCCATG
		Reverse	GATCCACACTCTCCAGCTGCA
	IL6	Forward	ACCAGAGGAAATTTTCAATAGGC
		Reverse	TGATGCACTTGCAGAAAACA
	IL23p19	Forward	CCAGCGGGACATATGAATCT
		Reverse	AGGCTCCCCTTTGAAGATGT
	CXCL13	Forward	AACTCCACCTCCAGGCAGAATG
		Reverse	TGTGTAATGGGCTTCCAGAATACC
	CCL21	Forward	GCAAAGAGGGAGCTAGAAAACAGA
		Reverse	TGGACGGAGGCCAGCAT
	CXCR5	Forward	GACTCCTTACCACAGTGCACCTT
		Reverse	GGAAACGGGAGGTGAACCA

Supplemental Table 6. Primers for RT-qPCR analyses.
